# MSLibrarian: Optimized Predicted Spectral Libraries for Data-Independent Acquisition Proteomics

**DOI:** 10.1021/acs.jproteome.1c00796

**Published:** 2022-01-19

**Authors:** Marc Isaksson, Christofer Karlsson, Thomas Laurell, Agnete Kirkeby, Moritz Heusel

**Affiliations:** † Department of Biomedical Engineering, Lund University, 22100 Lund, Sweden; ‡ Department of Experimental Medical Science and Wallenberg Center for Molecular Medicine, Lund University, 22100 Lund, Sweden; § Infection Medicine Proteomics Lab, Division of Infection Medicine (BMC), Faculty of Medicine, Lund University, 22100 Lund, Sweden; ∥ Department of Neuroscience, University of Copenhagen, DK-2200 Copenhagen, Denmark; ⊥ The Novo Nordisk Foundation Center for Stem Cell Biology (DanStem), Faculty of Health and Medical Sciences, University of Copenhagen, DK-2200 Copenhagen, Denmark

**Keywords:** data-independent acquisition, spectral predictions, proteomics, deep-learning, R-software

## Abstract

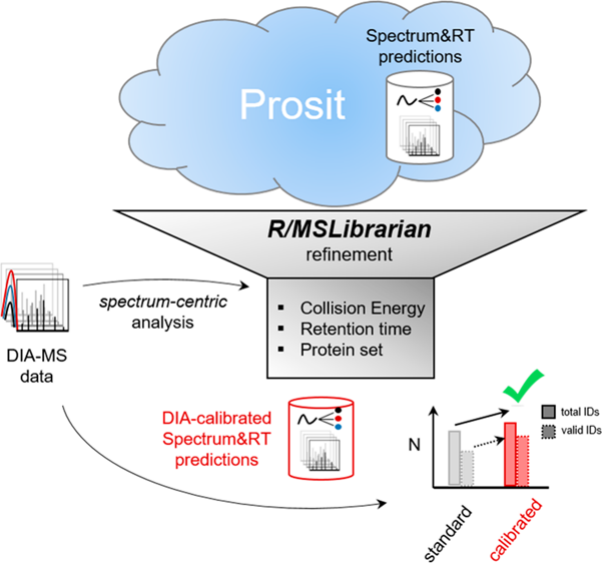

Data-independent acquisition-mass spectrometry (DIA-MS) is the method of choice for deep, consistent, and accurate single-shot profiling in bottom-up proteomics. While classic workflows for targeted quantification from DIA-MS data require auxiliary data-dependent acquisition (DDA) MS analysis of subject samples to derive prior-knowledge spectral libraries, library-free approaches based on *in silico* prediction promise deep DIA-MS profiling with reduced experimental effort and cost. Coverage and sensitivity in such analyses are however limited, in part, by the large library size and persistent deviations from the experimental data. We present MSLibrarian, a new workflow and tool to obtain optimized predicted spectral libraries by the integrated usage of spectrum-centric DIA data interpretation via the DIA-Umpire approach to inform and calibrate the *in silico* predicted library and analysis approach. Predicted-vs-observed comparisons enabled optimization of intensity prediction parameters, calibration of retention time prediction for deviating chromatographic setups, and optimization of the library scope and sample representativeness. Benchmarking via a dedicated ground-truth-embedded experiment of species-mixed proteins and quantitative ratio-validation confirmed gains of up to 13% on peptide and 8% on protein level at equivalent FDR control and validation criteria. MSLibrarian is made available as an open-source R software package, including step-by-step user instructions, at https://github.com/MarcIsak/MSLibrarian.

## Introduction

Mass spectrometry-based proteomics allows for the quantification of thousands of proteins in a single sample.^1^ Especially, data-independent acquisition (DIA) of mass spectra allows for reproducible protein quantification with few missing values.^2,3^ In classical, targeted peptide-centric DIA analysis,^2,4^ a spectral library is required to identify peptides from the highly convoluted DIA-MS data. Such a spectral library is commonly built from data-dependent acquisition (DDA) runs of prefractionated experiment-specific samples.^5,6^ Despite the benefits of building a sample-specific and deep library, this approach requires an additional sample amount, preparation, and MS run time. Also, as the semistochastic DDA method selects the most abundant precursors for sequencing, precursors of low abundant proteins might go undetected. As an alternative to experimental DDA spectral libraries, two classes of library-free approaches to the interpretation of DIA-MS data have been devised. First, spectrum-centric conversion approaches such as DIA-Umpire and Spectronaut directDIA^7,8^ convert the DIA- to a pseudo-DDA data structure for compatibility with classic spectrum-centric analysis workflows, based on grouping precursor and fragment ion signals from coelution and XIC signal correlation along the chromatographic dimension. While reaching high levels of proteomic coverage, conversion approaches so far do not reach the level of quantitative accuracy or proteomic depth achievable with dedicated, sample-specific libraries generated by DDA-MS.^8,9^ In contrast, recent applications of deep learning to predict MS/MS spectra and retention times now enable accurate *in silico* prediction of spectral libraries to support targeted, peptide-centric queries of virtually any peptide or precursor, extending DIA capabilities to previously uncharacterized proteomes.^10−12^ Similar to spectrum-centric-conversion approaches, predicted spectral libraries provide deep profiling in peptide-centric mining of DIA data sets, albeit not at the depth of dedicated and fractionated project-specific libraries. The prediction framework and Web server Prosit provides easy-to-use access to accurately predicted spectral libraries for DIA queries.^10^ In order to adapt and optimize fragmentation predictions to user’s MS instruments, users need to determine the optimal prediction parameter (normalized collision energy, CE) through provision of a set of identified DDA-MS spectra acquired with the same MS instrument and fragmentation parameters as the DIA data to be analyzed using the Prosit-predicted spectral library. The Prosit framework does currently not allow users to calibrate the retention time predictions to user-observed values. Three factors which limit the sensitivity of DIA-MS analyses via such *in silico* predicted libraries are (i) the prevailing inaccuracies of predicted spectra and/or stochasticity of observed fragmentation patterns, (ii) inaccuracies in expected retention times, as caused, *e.g.*, by the use of different chromatographic equipment or laboratory-to-laboratory variability, as well as (iii) the sheer library and search space size, resulting in a large number of tests and need for strict multiple hypothesis testing correction in statistical validation and FDR control.^6,13,14^

Here we present MSLibrarian, a workflow building on the Prosit,^10^ DIA-Umpire,^8^ and DeepLC^11^ frameworks, allowing one to obtain optimized predicted spectral libraries for DIA proteomics. This is related to existing predicted spectral library refinement strategies that rely on gas-phase fractionated, narrow isolation window DIA measurements and chromatogram libraries^15^ or which reuse empirically observed retention times and fragmentation patterns in multipass analyses.^16^ In contrast to these, MSLibrarian leverages a spectrum-centric conversion and analysis approach to optimize parameters for the prediction of both fragmentation patterns as well as chromatographic retention time directly based on the DIA data set being analyzed. In addition, MSLibrarian employs variable FDR multipass analysis to constrain the target protein set and thereby improve the library representativeness, as well as library size optimization by fragment ion selection. We quantified the improvements for predicted library-based DIA-MS data analysis incurred by MSLibrarian calibration based on a dedicated ground-truth-embedded species mixture experiment and additional data sets and tools. The MSLibrarian R package, with step-by-step user instructions, is available at https://github.com/MarcIsak/MSLibrarian.

## Materials and Methods

### Generation of a Ground-Truth Mixed Species Proteome Data Set

To benchmark prospective improvements for quantitative DIA-MS data analyses via MSLibrarian library calibrations, we generated a simplistic two-species mixture data set composed of tryptic peptides derived from mouse spleen and yeast full proteomes. Mouse spleens were harvested from 12-week-old female C57BL/6J mice and then homogenized in PBS (Gibco) using a bead-beater (MP-Biomedicals). Proteins were extracted and denatured with 8 M urea in 0.1 mM ammonium bicarbonate (Sigma-Aldrich), and debris was remove by centrifugation at 14,000 *g* for 5 min. Cysteines were reduced using 50 mM tris(2-carboxyethyl)phosphine (Sigma-Aldrich) and then alkylated with 100 mM iodoacetamide (Sigma-Aldrich). Protein concentration was determined with the bicinchoninic acid assay (Thermo Scientific). Protein extract (50 μg) was digested with 1 μg of sequencing grade modified trypsin (Promega). The resulting peptides were desalted with C18 reversed-phase chromatography (ultramicrospin-columns) according to the manufacturer’s instructions (Harvard Apparatus). The mouse peptides were dried down with a vacuum concentrator (Savant). Yeast tryptic peptides, from *Saccharomyces cerevisiae* whole-cell protein extract, were purchased from Promega (Promega catalog no. V7461). Mouse and yeast peptides were resuspended in 0.1% formic acid, 2% acetonitrile in water at a concentration of 1 μg/μL and mixed at a ratio of 6:1 v/v mouse–yeast for samples of condition A and 1:6 v/v mouse–yeast of condition B. A volume of 1 μg of the mouse–yeast hybrid proteomes were analyzed with DIA-MS in technical reinjection triplicates per each of the two conditions A and B.

### Data-Independent Acquisition MS Analysis of Mouse–Yeast Hybrid Samples

All peptide analyses were performed on a Q Exactive HF-X mass spectrometer (Thermo Fisher Scientific) connected to an EASY-nLC 1200 ultrahigh-performance liquid chromatography system (Thermo Fisher Scientific). Peptides were trapped on the precolumn (PepMap100 C18 3 μm; 75 μm × 2 cm, Thermo Fisher Scientific) and separated on an EASY-Spray column (ES803, column temperature 45 °C, Thermo Fisher Scientific). Equilibrations of columns and sample loading were performed per manufacturer’s guidelines. Solvent A was 0.1% formic acid, and solvent B (0.1% formic acid, 80% acetonitrile) was used to run a linear gradient from 5 to 38% over 120 min at a flow rate of 350 nL/min. The mass range for MS1 was 350–1 650 *m*/*z* with a resolution of 120,000 and a resolution of 30,000 for MS/MS with stepped normalized collision energies (NCE) of 25.5, 27, and 30. The data-independent acquisition (DIA) method was derived from Bruderer *et al.*(17)). The 44 variably sized MS/MS windows were 350–371, 370–387, 386–403, 402–416, 415–427, 426–439, 438–451, 450–462, 461–472, 471–483, 482–494, 493–505, 504–515, 514–525, 524–537, 536–548, 547–557, 556–568, 567–580, 579–591, 590–603, 602–614, 613–626, 625–638, 637–651, 650–664, 663–677, 676–690, 689–704, 703–719, 718–735, 734–753, 752–771, 770–790, 789–811, 810–832, 831–857, 856–884, 883–916, 915–955, 954–997, 996–1057, 1 056–1 135 and 1 134–1 650 *m*/*z*, resulting in a total cycle time of ∼3.3 s and 6–8 sampling points per chromatographic peak on average.

### Data-Dependent Acquisition MS Analysis

A representative sample of the mouse spleen proteome was analyzed by DDA mass spectrometry to generate identification results to calibrate the Prosit prediction model with a fixed CE value along the canonical workflow for Prosit predictions.

For data dependent acquisition, one full MS scan (resolution 60,000 at 200 *m*/*z*; mass range 350–1650 *m*/*z*) was followed by MS/MS scans (resolution 15,000 at 200 *m*/*z*) of the 20 most abundant ion signals. The precursor ions were isolated with a 1.6 *m*/*z* width and fragmented using higher-energy collisional-induced dissociation at a normalized collision energy of 27. Charge state screening was enabled and unassigned or singly charged ions were rejected. The dynamic exclusion window was set to 15 s. Only MS precursors that exceeded a threshold of 8 × 10^3^ were allowed to trigger MS/MS scans. The ion accumulation time (IT) was set to 100 ms (MS) and 30 ms (MS/MS) using an automatic gain control (AGC) target setting of 2 × 10^5^ (MS and MS/MS).

### Determination of CE for Spectral Library Predictions with the Prosit Online Tool

The acquired DDA-MS file, as described in the previous section, was imported into RecalOffline (Build No. 3.0.0.19, Thermo Fisher) and sliced to only include the first 100 min of the LC gradient. The slicing was made to acquire a file smaller than 2 GB, a restriction imposed by the Prosit CE calibration online tool. Subsequently, the sliced DDA-MS file was loaded into MaxQuant (v.1.6.1.0) to be searched with trypsin as the enzyme, LFQ disabled, no modifications except carbamidomethyl (C) set as a fixed modification. A protein sequence FASTA, created from the canonical mouse proteome (*Mus musculus*, UniProt/Swiss-Prot release 2021_03), was configured as a sequence database. Identification settings were left at the default, with an FDR = 1% on PSM and protein level, and the second peptide search was disabled. Once the MaxQuant search was finished, the resulting msms.txt file and the sliced DDA-MS file were uploaded to the Prosit server according to the instructions for the online CE calibration tool. Similarly, a representative DDA-MS file was sliced in RecalOffline and used to determine an optimal CE value spectral library predictions for the external mixed species data set (PXD005573). MaxQuant parameters were the same as for the sliced DDA-MS file for the mouse-spleen sample described above. A merged protein sequence FASTA, created from the individual FASTA files of the canonical proteomes for human, yeast, *C. elegans*, *E. coli* strain K12 (UniProt release 2021_03, canonical sequences, UP000005640, UP000002311, UP000001940, and UP000000625), was used for the MaxQuant search.

### Spectrum-Centric DIA-MS Data Analysis

As an integral part of the MSLibrarian workflow, DIA-MS data were first analyzed via the DIA-Umpire workflow to convert DIA data structures to pseudo-DDA (pDDA) spectra (MSconvert, ProteoWizard release 3.0.20365) via the implemented DIA-Umpire signal extraction module, operated with standard parameters). pDDA spectra were then searched against the respective protein sequence database via MSFragger (v3.2) where the default parameter file (*.params) for closed searches was used as a template with trypsin as the enzyme, precursor charges of 2 and 3, and peptide lengths from 7 to 30 AA allowed. Carbamidomethylation on cysteines was set as the fixed modification but no variable modifications. The MSFragger output pep.xml-files were imported into PeptideProphet (TPP v.5.2.0) for PSM validation using the nonparametric model with decoys modeling the negative PSM distribution. The retention time model and accurate mass binning options of PeptideProphet were enabled. The resulting *pep.xml files were then imported into iProphet (TPP v.5.2.0) for further PSM validation. Only PSMs having a posterior error probability (local FDR) ≤ 0.01 were used for consensus spectral library creation with Spectrast (TPP v.5.2.0) to ensure high spectral quality. A set of 11 iRT peptides with known iRT values (Biognosys, iRT kit) were used by Spectrast to convert RT in seconds to iRT values. OpenSwathAssayGenerator (OpenMS v2.5.0) was then used to convert the consensus.splib library format into the MSLibrarian-compatible OpenSwath (.TSV) format to be used as a latter calibration library, ready for comparisons to the predicted spectra per each matched peptide precursor ion. Spectrum-centric search and assembly of results into the calibration library are accessible in MSLibrarian via the function *create.calibration.lib* (Figure S1, top left).

### Spectrum-Spectrum Matching and Library Formulation in MSLibrarian

Spectrum-centric identifications from the DIA data were matched to spectral warehouse database entries via the MSLibrarian functions process.calibration.lib and create.spectral.lib (Figure S1, lower left and upper right panel). For each precursor length and charge bin, spectra predicted with collision energies resulting in maximal similarity, as measured by the dot product score, are selected for inclusion into the spectral library produced via the function *create.spectral.lib*. The product library contains precursor length- and charge-dependent, variable collision energies (CE-LZ). Further processing steps are executed via the function mod.spectral.lib and include (i) retention time replacement with calibrated DeepLC predictions, (ii) protein group subsetting to a list of proteins from first-pass DIA-NN analysis with relaxed FDR criterion (5% protein-level FDR), and (iii) subsetting of transitions/fragment ions to be included in the final library (library variants, _RT, _Pr and _Tr, respectively) (Figure S1, bottom right).

### Peptide-Centric DIA-MS Data Analysis

For peptide-centric analyses of the DIA data sets, leveraging either of the compared spectral libraries as prior information for the analysis, DIA-NN (v1.8)^18^ and EncyclopeDIA (v1.2.2)^19^ tools were employed. DIA-NN operated directly on the Thermo.raw files, whereas EncyclopeDIA analysis commenced from .mzML format upon conversion via MSconvert (Centroiding the MS1 level via the peak picking option, ProteoWizard release 3.0.20365). DIA-NN was run with fixed MS1 accuracies (4.92 and 3.93 ppm for the mouse-yeast data set and external mixed species data set, respectively) based on recommended MS1 values by DIA-NN from first pass analysis of the samples with automated determination of optimal mass accuracies. The MS/MS accuracy was automatically determined by DIA-NN as a consequence of the set fixed MS1 accuracies (MS/MS accuracy = 2 × 10^–5^, *i.e.*, 20 ppm, for both data sets). The retention time extraction window was determined individually for all MS runs analyzed via the automated optimization procedure implemented in DIA-NN. Protein inference was enabled, and the quantification strategy was set to Robust LC = High Accuracy. The precursor-level FDR was set to 1%. The flag report-lib-info was set in order to report fragment-level intensities for quantification in the R package iq.^20^ Output main DIA-NN reports were filtered with a global FDR = 0.01 on both the precursor level and protein group level. Quantitative matrices on the peptide and protein group and gene level were extracted from the main DIA-NN reports using the diann-rpackage (https://github.com/vdemichev/diann-rpackage). For fragment-level MaxLFQ analysis via iq,^20^ fragment ion information associated with each entry in the main DIA-NN reports (intensities from column *Fragment.Quant.Corrected*) was extracted and concatenated to its precursor, protein group, and MS run. Note that for the mouse-yeast data set, where raw intensities in the absence of further processing were of interest, values from column *Fragment.Quant.Raw* were used. Fragment ions with a log2-intensity ≤ 0 were removed. Median normalization in iq was applied to the external mixed species data set, while no normalization was applied to the mouse-yeast data set. MaxLFQ-based pairwise ratio estimation between common fragment ions for the same precursors of each protein group was performed. In the last step, a full quantitative intensity matrix with protein groups as row headers and MS-runs as column headers was written out. Similarly, a quantitative matrix for stripped peptide sequences was generated, where fragment ions and precursors were grouped based on peptide sequences.

For the EncyclopeDIA analyses, all generated spectral libraries were first converted from the Spectronaut format (.CSV) to the DLIB-format. Following library conversions, DIA-MS files were analyzed sequentially for each library using default settings and with HCD as fragmentation. A global EncyclopeDIA analysis was then carried out to retrieve quantitative matrices for peptides and protein groups identified with a FDR ≤ 0.01. Median normalization was applied to the quantitative matrices using the R-package NormalyzerDE.^21^

### Generation of Spectral Warehouse Databases

Spectral warehouse databases have been precomputed as described below for the following proteomes: *H. sapiens*, *M. musculus*, *S. cerevisiae*, *C. elegans*, *E. coli strain K12*, and *D. melanogaster* (UniProt/Swiss-Prot release 2021_03, canonical and isoform sequences for proteome IDs UP000005640, UP000000589, UP000002311, UP000001940, UP000000625, and UP000000803). The precomputed databases can be downloaded from Zenodo by users of MSLibrarian with the function *get.spectral.db* (Figure S1). This obviates the need for users to use the Prosit Web-services or set up local instances of the Prosit predictor for these most common usage scenarios.

Spectral warehouse databases were generated from *in silico* predicted peptide fragmentation spectra along collision energies ranging from 20 to 40, using the Prosit 2020 HCD model with built-in retention time prediction (https://www.proteomicsdb.org/prosit/).^10^ The results, 21 variant fragment intensity sets and one iRT value per precursor, were stored in a nonredundant SQL database, storing the multi-CE intensity sets in an efficient manner. Detailed step-by-step instructions to assemble warehouse predictions for custom protein sequence databases are included in the MSLibrarian usage wiki on Github (https://github.com/MarcIsak/MSLibrarian/wiki/).

### MSLibrarian-Based CE Calibration

Each calibration library in OpenSwath format (*.TSV) was imported into MSLibrarian, where library entries were filtered to only contain predictable precursors with lengths of 7–30 amino acids and charge states 2 and 3. Remaining precursors were mapped to precursor entries in the spectral warehouse database. MS/MS information, for precursors in the calibration library and matching precursors in the spectral warehouse database, was extracted to create Spectrum2 objects using the R software package MSnbase.^22,23^ Spectral comparisons were carried using the dot product score, as implemented in the MSnbase library, as a metric for spectral similarity between experimental spectra and predicted spectra. The comparisons were performed for all predicted spectra with CE values in the range of 20–40.

### Retention Time Prediction via DeepLC

Peptide retention times in reversed-phase chromatography were predicted using DeepLC^11^ (v.0.1.29). Retention time prediction was calibrated based on experimental retention times (represented in iRT values) of 25% of all targets in the spectrum-centric search-based calibration libraries created by MSLibrarian. Using this calibrated model, iRT values for all targets in the spectral libraries were predicted and introduced into the libraries via the MSLibrarian function *mod.spectral.lib* replacing the native Prosit-predicted iRT values with the calibrated iRT values from DeepLC for each target in the respective library (Figure S1, lower right).

### Benchmarking through Quantitative Validation

To compare the different libraries’ performance in generating correct identifications and quantifications from the DIA data sets, quantitative matrices as generated by DIA-NN in conjunction with the diann-rpackage or the iq package and EncyclopeDIA for both peptides and protein groups were processed in R. Peptide and protein detections were validated based on conformance with the known species mixing ratio embedded in the ground truth data sets. Identifications supported by an observed quantitative ratio value within a tolerance of ±20% from the expected ratio on the linear scale were considered valid. Accordingly, each library produced a certain number of high-quality identifications on the peptide and protein group levels that were supported by matching quantitative ratios in the sample group comparison. The number of valid values were calculated for both, protein group and peptide levels in the separate matrices. Protein quantification data together with valid ratio Boolean are provided in Table S1.

### MSLibrarian R Package Development and Availability

The MSLibrarian workflow has been implemented as an R software package using devtools and roxygen2 libraries. Dependencies from the R ecosystem include tidyverse, stringr, readr, dplyr, and ggplot2. MSLibrarian has been developed and tested in Windows environments. Additionally, MSLibrarian depends on third party software utilized within the workflow, specifically, MSconvert (as part of the ProteoWizard suite of tools, https://proteowizard.sourceforge.io/),^24^ the Trans-Proteomic Pipeline (TPP, https://sourceforge.net/projects/sashimi/files/Trans-Proteomic%20Pipeline%20%28TPP%29/),^25^ OpenMS (https://github.com/OpenMS/OpenMS/releases/tag/Release2.6.0), MSFragger (https://github.com/Nesvilab/MSFragger,^26^ DeepLC (https://github.com/compomics/DeepLC),^11^ and DIA-NN (https://github.com/vdemichev/DiaNN/releases/tag/1.8). Full details on how to download, install, and run MSLibrarian can be found at the package Github repository and Wiki page (https://github.com/MarcIsak/MSLibrarian). Raw data, peptide-centric analysis results as well as spectral warehouse databases, spectral libraries, and protein sequence databases have been uploaded to the ProteomeXchange Consortium via the Pride partner repository (accession number: PXD028901).

## Results and Discussion

### Guiding Principles of the MSLibrarian Workflow

The MSLibrarian workflow builds on Prosit *in silico* predicted spectral libraries and refines these for improved performance in downstream targeted, peptide-centric DIA analyses. Refinement and optimization via MSLibrarian is based on three key principles:

(I) Peptide fragmentation is subject to instrument- and run-by-run variability and differs in narrow isolation-window DDA when compared to wide-isolation-window DIA operation of the mass spectrometer. MSLibrarian optimizes the Prosit CE prediction parameter directly comparing against the DIA data to be analyzed using the library, and in a peptide charge state- and -length-dependent manner and using the dot product as a metric. To this end, rather than trying different prediction parameters “on the fly”, MSLibrarian relies on fragment ion intensities predicted across a range of collision energy parameter settings *a priori*, efficiently stored in a ‘spectral warehouse’.sql database and available for multiple species (see Materials and Methods section).
(II) Peptide retention time and accuracy of its prediction depend on training data and LC equipment, such as bead pore size and chemistry, used in each laboratory. MSLibrarian negotiates prediction of more accurate retention times, calibrating retention time prediction based on observed values in the DIA data set and an underlying liquid chromatography setup. Notably, a conservative and deliberate decision not to (re-) use the empirically observed retention time values of DIA-Umpire-identified precursors in subsequent peptide-centric analyses was made. We reason that this choice is conservative as it counteracts potential leakage of false positive spectrum-centric identifications into the peptide-centric analysis results because of artificially low retention time offsets for a subset of target peptides where spectrum-centric evidence is available.
(III) Single-shot DIA measurements with a gradient time below 2 h likely do not contain more than 100,000 detectable precursor signals due to technological constraints, such as limited intrascan dynamic range.^27^ Thus, querying millions of predictable precursors per proteome unnecessarily escalates the need for multiple testing correction in statistical FDR control and, thereby, limits the sensitivity and coverage of such analyses. It is thus beneficial to trim the library to a more relevant set of precursors containing those detectable in the DIA-MS record while not compromising discovery by removing relevant ones.^14^ To this end, MSLibrarian implements a two-tiered library representativeness optimization approach based on multipass variable FDR analysis and transition filtering.

### Steps of the MSLibrarian Workflow

**Figure 1:**
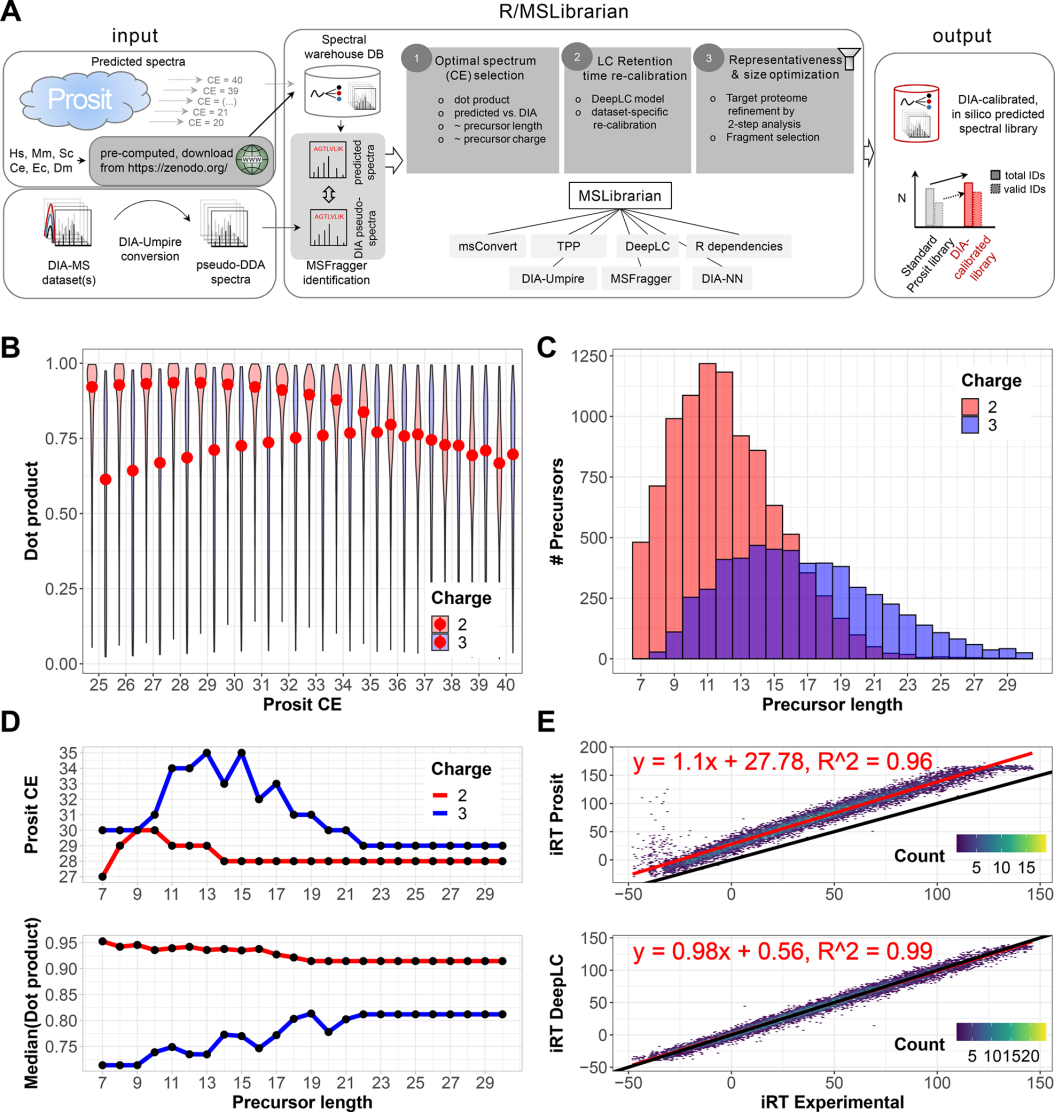
MSLibrarian workflow for DIA-guided predicted library optimization. (A) Schematic overview of the MSLibrarian workflow, taking as input fragmentation spectra predicted using Prosit along a range of CE settings and stored in a spectral warehouse SQLite database and DIA data to be analyzed. DIA-MS data are converted to pseudo-DDA spectra, and these are identified via spectrum-centric search and fragment intensities compared against predicted spectra. MSLibrarian then (1) selects optimal collision energy spectra per precursor length and charge group, (2) recalibrates LC retention time by DeepLC calibration and prediction, and (3) optimizes library representativeness by variable FDR multipass analysis and fragment selection. (B) Exemplary distribution of dot product scores for a representative peptide group of length 15, indicating divergent optimal prediction CE parameter settings per charge state. In this example, the optimal CE parameters are 28 and 35 for charge states 2 and 3, respectively, as shown by the median dot product (red dots). (C) Number of precursors per peptide length group. (D) Exemplary set of optimal CE parameter settings per peptide length and charge and optimal median dot product achieved. (E) Example of LC normalized retention time recalibration to DIA data set generated on different LC-MS setups in comparison to uncalibrated retention times.

A schematic overview of the MSLibrarian workflow is shown in Figure 1A. Inputs to the workflow are spectrum predictions from Prosit stored in a spectral warehouse database, accompanied by the corresponding protein sequence database, and a DIA-MS data set.

In the first step, predicted spectra are obtained using the Prosit Web server (see Materials and Methods). Note that for frequently studied proteomes (*H. sapiens*, *M. musculus*, *S. cerevisiae*, *C. elegans*, *E. coli*, and *D. melanogaster*), precomputed spectral warehouse databases can be downloaded from Zenodo.org from within MSLibrarian (Figure 1A, left).

In the second step, DIA-MS data are converted to pseudo-DDA (pDDA) spectra which are then identified by a spectrum-centric search against the protein sequence database (Figure 1A, left).

In the third step, the identified spectra are then compared to the set of spectra predicted for this precursor across the range of CE prediction parameter values (stored in the spectral warehouse database) using the dot product score. Comparisons are binned by peptide length and precursor charge state, producing dot product distributions as exemplified in Figure 1B for typically ∼100–1200 precursors per comparison bin (Figure 1C). For each length and charge bin, spectra predicted with collision energies resulting in maximal similarity, as measured by the dot product score, are selected for inclusion into the MSLibrarian library (Figure 1D and Materials and Methods).

In the fourth step, peptide retention times are predicted via the DeepLC tool, with calibration based on the retention times of peptides identified via spectrum-centric analysis, effectively adjusting parameters for deviating chromatographic setups (Figure 1E).

In the fifth step, the set of detectable proteins is estimated by a reduced stringency peptide-centric analysis of the DIA data with the MSLibrarian library in its current state using DIA-NN, noting all target protein groups identified at an increased FDR threshold of 5% on the protein level. In addition, the product library is trimmed, retaining only the 6–14 most-intense fragment ion signals of a given spectrum (Figure 1A, center). The workflow produces as output a refined version of the input *in silico* predicted spectral library, calibrated for preferable performance in peptide-centric analysis of the given DIA-MS data set (Figure 1A, right).

### Benchmarking DIA-Guided Library Calibration via MSLibrarian

**Figure 2:**
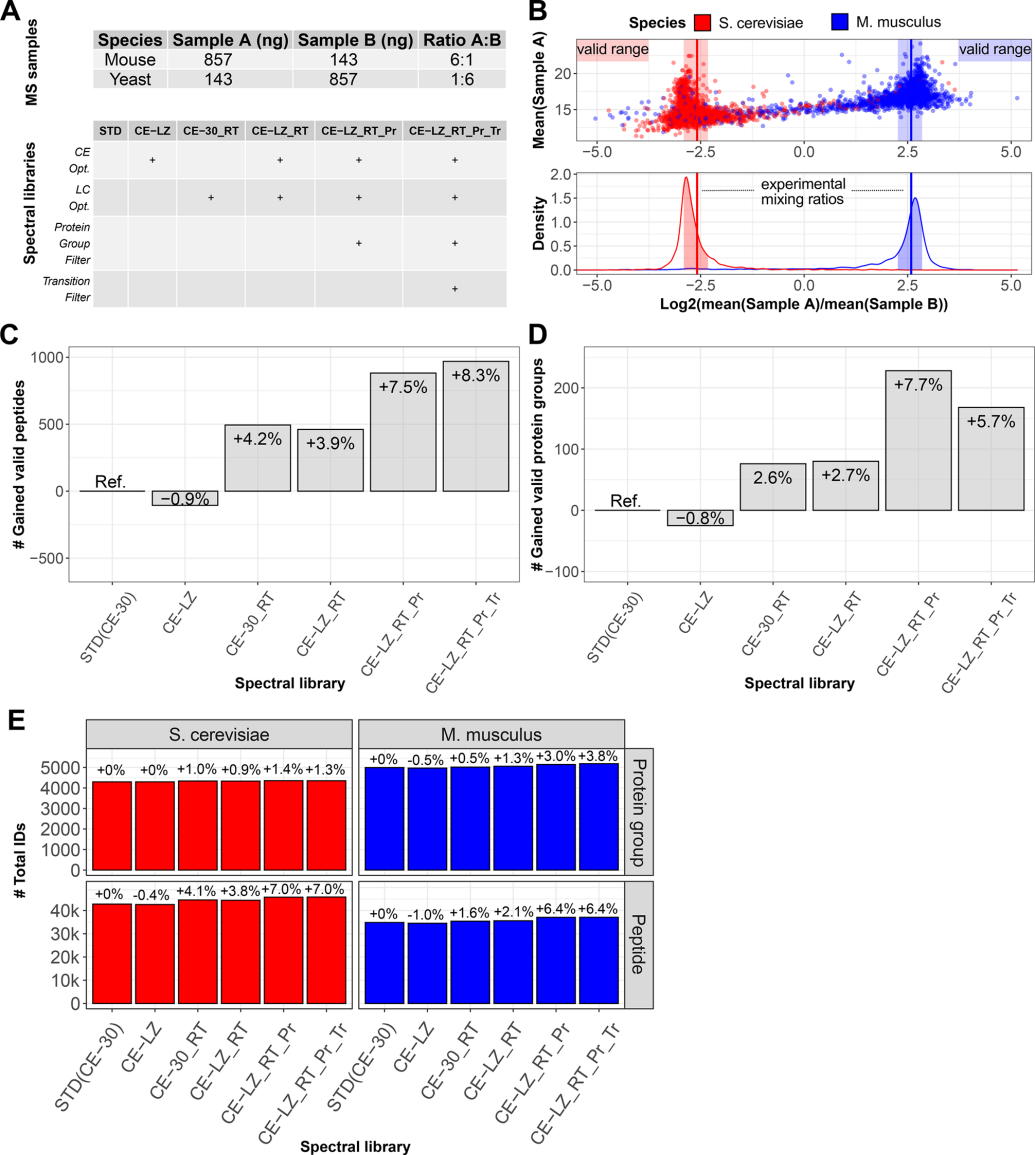
Benchmarking DIA-guided library calibration via MSLibrarian. (A) Experimental design of the two-species proteome sample set analyzed in triplicate DIA-MS injections (upper panel) and schematic overview of processing steps included to generate each of the spectral libraries in the present comparison (lower panel). (B) Observed protein level quantitative ratios across the abundance range and represented as density distributions for library CE-LZ_RT_Pr_Tr. The range in which quantitative values were considered valid is highlighted as shaded areas. (C) Number of ratio-validated analytes detected via either library, quantifying changes relative to using the standard Prosit library with CE calibration on the DDA-MS data set, for peptides. (D) Equivalent to panel C but on the protein group level after protein quantification via fragment-level MaxLFQ via R/iq (Materials and Methods). (E) Overview of total peptide and protein identification numbers and changes relative to STD (CE-30) achieved via either library.

To validate the impact of library refinements through MSLibrarian and to ensure these were not noise from false positive identifications, we generated a simple two-proteome species mixture proteomic data set with a known ground truth embedded as defined proteome mixing ratios, along the LFQbench rationale.^9^ Specifically, we acquired DIA data of a mouse-yeast tryptic peptide mixture with a stable amount of mouse and yeast tryptic peptides mixed to obtain a ratio of 6:1 for mouse peptides and 1:6 for yeast peptides in the comparison of sample A to sample B (Figure 2A and Materials and Methods). Considering only identifications that were validated by conformance of the observed quantitative ratio with the true mixing ratio allowed us to compare the libraries’ proteomic profiling efficiency, with auxiliary confidence in the correctness of the assigned signals and validity of observed gains. As a reference baseline of the comparison, the canonical Prosit-derived library workflow was applied, obtaining a full proteome predicted library with a fixed collision energy setting, calibrated based on a side-by-side DDA-MS raw file and accompanying MaxQuant search results (Fixed CE = 30, “STD-Prosit” Library, Materials and Methods). In order to assess the impact of the individual library refinement steps applied by MSLibrarian, partially optimized libraries gradually including more refinement steps toward the full MSLibrarian-calibrated library were included. Specifically, library CE-LZ included only collision energy parameter optimizations affecting fragment intensities; library CE-30_RT included only retention time prediction recalibration via DeepLC; library CE-LZ_RT combined both variable collision energy parameter optimization and retention time prediction recalibration; library CE-LZ_LC_Pr added protein set confinement; and the final, fully MSLibrarian-calibrated library CE-LZ_RT_Pr_Tr added fragment selection (schematized in Figure 2A, bottom panel). The libraries were then searched against the DIA-MS data by peptide-centric analysis via DIA-NN and peptides and proteins quantified via the iq package^20^ that implements the MaxLFQ approach to calculate the relative protein group and peptide quantities for all samples. Cross-sample ratios were calculated on both the peptide and protein levels separately, and identifications with quantitative ratios conforming with the known mixing ratio within the ±20% tolerance were considered valid in the primary benchmark (Figure 2B, Table S1, and Materials and Methods). At the peptide level, up to 8% improvement was observed, with library modifications ranking by decreasing benefit for peptide-level performance as follows: protein set confinement > LC calibration > transition selection (Figure 2C). Variable CE selection, in this data set, was detrimental to peptide-level performance, whereas this was not the case on the protein level. On the protein level, a similar improvement of up to 7.7% additional protein groups (henceforth also referred to as proteins) detectable with valid quantitative ratios was observed, albeit only in the absence of transition filtering that had a detrimental impact on the fidelity of protein quantities in this data set (Figure 2D). Having confirmed that more ratio-conformant, valid quantifications were produced when employing refined libraries in peptide-centric processing, we moved on to compare the total identification numbers reported at equivalent FDR control per analysis (global protein and precursor level FDR 1%). Overall, up to 7% more peptides and 3% more proteins (9534 vs 9287) were identified using the MSLibrarian-calibrated library relative to the standard Prosit library (Figure 2E). Overall, MSLibrarian-based calibration of predicted libraries, in our hands, led to noticeable improvements in peptide-centric DIA-MS analyses. Notably, library improvements resulted primarily in improved quantification as observed by ratio validation analysis, with more modest gains in total identification numbers irrespective of quantitative conformance criteria (Figure 2E). An overview of observed species ratios and ratio distributions using the different libraries are visualized in Figure S2, and protein quantitative information is summarized in Table S1.

### Performance with External Data Set and Alternative Analysis Tool

**Figure 3:**
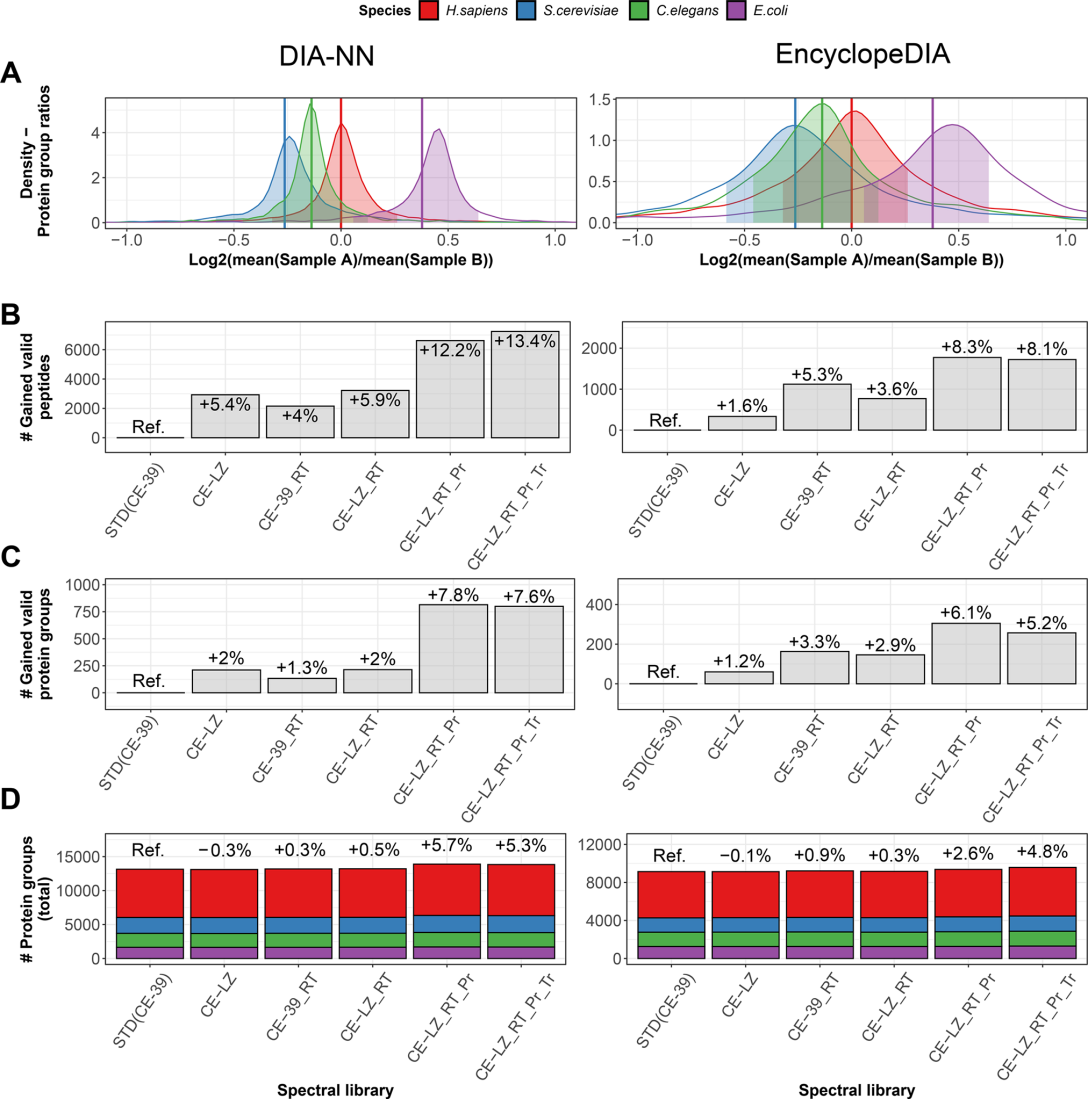
Benchmarking on 4-species data set with DIA-NN and EncyclopeDIA as downstream analysis tools. Left column: Results from DIA-NN. Right column: Results from EncyclopeDIA. (A) Overall intensity ratio distributions observed for the four species in the data set from Bruderer *et al.*, on protein level, employing the fully optimized library CE-LZ_RT_Pr_Tr. (B) Number of ratio-validated peptide analytes gained by employing either library, quantifying changes relative to using the standard Prosit library with CE calibration on the DDA-MS data set. (C) Number of ratio-validated peptide analytes gained by employing either library, quantifying changes relative to using the standard Prosit library with CE calibration on the DDA-MS data set. (D) Overview of total peptide and protein identification numbers achieved via either library and downstream peptide-centric analysis tools.

Next, we explored whether the benefits of DIA-based calibration of predicted spectral libraries observed in our internal benchmark experiment could also be replicated on an external data set using an alternative software framework for targeted, peptide-centric analysis with these libraries as prior knowledge. We selected a high complexity data set generated by Bruderer *et al.*(17) with a similar multispecies mixture setup, containing a total of four proteomes with only small mixing ratio differences between the two sample sets comprising the “low ratio” LFQbench data set in the study. We then analyzed this data set via the standard Prosit workflow, calibrating the CE parameter based on the side-by-side DDA-MS measurement and MaxQuant search results to obtain the standard Prosit library. As described above, we comparatively processed the data set with the MSLibrarian workflow, in part and in full, to generate the partially processed as well as fully processed DIA-calibrated library (compare scheme in Figure 2A, bottom panel). The DIA data were then analyzed by peptide-centric analysis by DIA-NN as above as well as using EncyclopeDIA^19^ as an alternative software (For parameters, see the Materials and Methods). Identifications per species were then categorized based on validity of their quantitative ratios, relative to the true mixing ratio with ±20% tolerance, equivalent to the benchmark used for the internal data set presented above (exemplified in Figure 3A).

Comparing the standard Prosit library STD (CE-39) and the MSLibrarian-processed libraries, peptide level gains of up to 13.4% (>7000 peptides) and 8.3% (>1500 peptides) were achieved in combination with downstream processing via DIA-NN or EncyclopeDIA, respectively (Figure 3B and Table S1). On the level of protein groups passing the validation criteria, processed libraries achieved gains of up to 7.8% (>750 proteins) and 6.1% (∼300 proteins) with DIA-NN and EncyclopeDIA, respectively (Figure 3C, Table S1). On the level of total identifications, gains of up to ∼5% on protein group level were observed for both DIA-NN and EncyclopeDIA (Figure 3D). For manual inspection, fold-change ratio distributions across the libraries not covered in Figure 3A are visualized in Figure S3A,B. Library optimization also affected peptide-centric analysis processing times, with optimized libraries allowing up to 40% shorter processing times evaluated for DIA-NN searches of the Bruderer *et al.* data set (Figure S5C).

### Simplified Workflow with Fixed Collision Energy Setting

Based on the observation that variable CE calibration was beneficial for library performance exclusively in the Bruderer *et al.* data set, we explored the impact of library optimizations on raw scores in peptide-centric analysis and evaluated the performance of an abbreviated MSLibrarian workflow that omits variable CE calibration. Based on the analyses via EncyclopeDIA, raw score distributions for both the internal mouse-yeast data set and the external Bruderer *et al.* data set were compared (Figure S5A,B). Retention time recalibration via DeepLC leads to sharper delta.RT score distributions and significantly lower absolute delta.RT readings consistently across replicates and both data sets (unpaired t.test *p* < 10 × 10^–4^ in all three replicates of both data sets, Figure S5A). Variable CE selection, comparing the library CE-LZ versus the respective standard, fixed CE library, leads to significantly higher Spectrum.Similarity scores in analysis of the Bruderer *et al.* data set, consistently across replicates (unpaired t.test *p* < 10 × 10^–4^ in all three replicates, Figure S5B). In the mouse-yeast data set, however, Spectrum.Similarity is reduced in two out of three replicates and is insignificantly higher in the third replicate (unpaired t.test *p* < 10 × 10^–4^ for reduced similarity in the first two replicates, Figure S5B). These observations indicate that the improvements by retention time recalibration more consistently benefit library quality than is the case for variable CE selection, which shows mixed results and therefore needs to be evaluated on a case-by-case basis.

Therefore, we assessed the performance of a workflow based on single, fixed CE, in combination with downstream optimization modules of MSLibrarian. Specifically, we generated libraries (fixed CE_Pr and fixed CE Pr_Tr) per each data set and compared their performance with the corresponding variable CE libraries (Figure S4 and see scheme in panel B). Indeed, fixed CE libraries, in combination with the MSLibrarian downstream processing (RT, Pr, Tr) exhibit good performance as judged by ratio-valid identifications along the benchmarking criteria introduced above (Figure S4A). On the peptide level, performance was indistinguishable from variable CE libraries in the mouse-yeast data set (maximal gain, 8.3% in both cases) and similar in the Bruderer *et al.* data set (11.8% vs 13.4% maximal gain with fixed vs variable CE calibration libraries, respectively) (Figure S4A). Protein level performance remained in most cases optimal when going the extra mile of variable CE selection (6.9% vs 7.7% maximal gain in mouse-yeast and 7.2% vs 7.8% maximal gain in the Bruderer *et al.* data set with fixed vs variable CE, respectively, Figure S4A). Total identifications remained essentially unchanged (Figure S4C). The shortened workflow based on fixed CE prediction, along the standard workflow or via a fixed CE approach in MSLibrarian directly on the DIA data, provided a viable entry point to optimization using the downstream modules of MSLibrarian with performance levels comparable to those of the full workflow with variable CE calibration.

### Impact of Library Optimization on Quantification Quality Metrics

To further explore the impact of MSLibrarian-based library optimization and the protein quantification strategy employed on the goodness of quantification, we used additional quality metrics relating to accuracy, precision, and variation of quantification in the external data set from Bruderer *et al.* Specifically, we measured (i) the precision of quantification based on the cumulative interquartile range of log2 fold change distributions observed across all species; (ii) the accuracy of quantification based on the cumulative absolute offsets of the observed log2 fold change distributions (represented by the most frequently observed value, *mode*) from the theoretical centers as per the known mixing ratios. In addition, offsets of % intensity change in linear space, equivalent to the analysis presented by Bruderer *et al.*,^17^ were employed as metrics for quantitative accuracy. Further, (iii) the coefficient of variation of protein group quantities within experimental replicates of the same biological condition was assessed.

**Figure 4:**
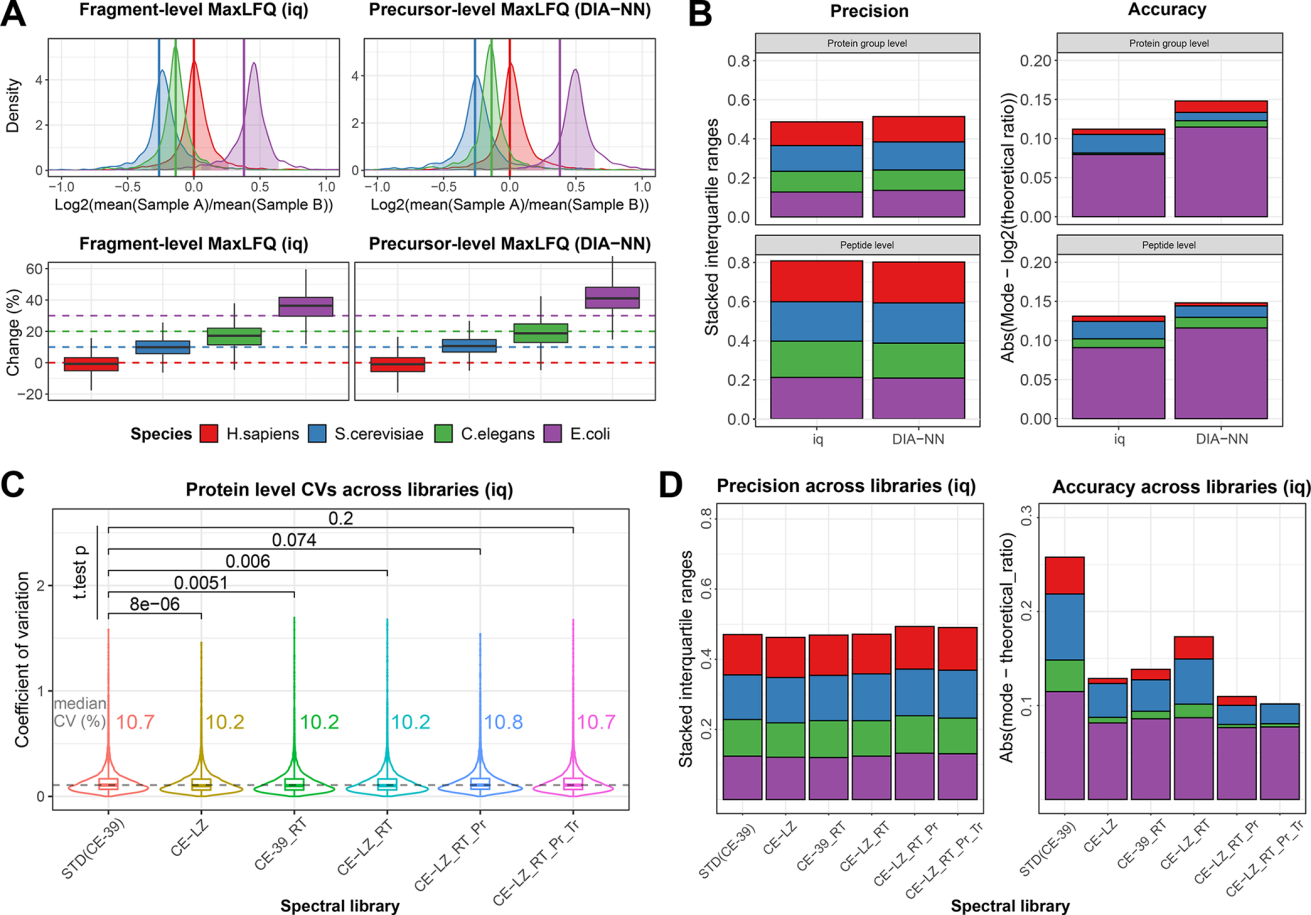
Impact of protein quantification and library optimizations on quantification quality metrics. (A) Impact of protein quantification strategy starting from fragment ion (left, iq) or precursor level (right, DIA-NN via the diann-rpackage), displayed as % change distributions in linear space as displayed in Bruderer *et al.*(17) and in log2 fold change ratio distribution space for reference. Data were generated via library CE-LZ_RT_Pr_Tr. (B) For the data displayed in panel A, precision and accuracy of quantification inferred through stacked species log2 fold change distribution interquartile ranges (left) and stacked offsets of most frequently observed ratio per species (Mode) from expected log2 fold change values (right) for both fragment-level and precursor-level MaxLFQ via iq or diann-rpackage. (C) Comparison of protein-level coefficients of variation upon fragment-level protein quantification across analyses performed with the differently processed libraries. Median CVs and statistical significance of these differences in paired *t* tests are indicated. (D) Precision and accuracy of protein quantification for the libraries displayed in Figure 3 and panel C based on fragment-level MaxLFQ via iq. Precision and accuracy of quantification inferred through stacked species log2 fold change distribution interquartile ranges (left) and stacked offsets of most frequently observed ratio per species (Mode) from expected log2 fold change values (right) across the libraries included in the main comparison.

First, we compared downstream protein quantification on precursor level (MaxLFQ, as implemented in DIA-NN) against quantification with ratio maximization on the fragment ion level (fragment-level MaxLFQ, as implemented in the R package iq), based on the analysis with the fully MSLibrarian-optimized library CE-LZ_RT_Pr_Tr. Both, log2 fold-change ratio distributions as well as % change distributions on a linear scale indicated higher accuracy of *E. coli* ratios and % change values (lower offset to theoretical value) when employing fragment-level ratio normalization via the iq package (Figure 4A, lower panels). When simplified via the interquartile ranges, precision appeared similar between the two methods, with a small advantage of the fragment-level procedure via iq (Figure 4B, left). However, quantification via iq clearly showed the benefits on the level of quantitative accuracy, indicated by lower overall offsets of the distributions from the theoretical ratios (Figure 4B, right).

Second, upon selection of the fragment-level ratio maximization strategy, we explored the impact of MSLibarian processing on the coefficient of variation of protein quantification across the different spectral libraries under investigation. Protein-level variation across libraries essentially remained stable, with only libraries CE-LZ, CE-39_RT, and CE-LZ_RT showing statistically significant reductions of observed CVs relative to the STD (CE-39) library (unpaired t.test *p*-values of 8 × 10^–16^, 5.1 × 10^–3^, and 6 × 10^–3^, respectively, Figure 4C). The further processed libraries including protein set optimization, CE-LZ_RT_Pr and CE-LZ_Pr_Tr, did not show significant changes of CV values (Figure 4C).

Third, we explored the precision and accuracy of quantification across the different libraries using the metrics as described above (Figure 4D). Precision of quantification remained essentially stable, with a minor negative impact of library processing (Figure 4D, left), whereas accuracy of quantification did improve with library processing (Figure 4D, right), suggesting that primarily improved accuracy, rather than precision, contributes to the observed gains of correctly quantified proteins upon MSLibrarian optimization.

## Conclusions

Predicted spectral libraries promise deep mining of DIA data sets independent of side-by-side DDA-MS analyses, yet with limited sensitivity of such analyses due to residual differences between predictions and the signals in the DIA data and large library search space.

Here, we present an approach to calibrate predicted spectral libraries directly based on the DIA-MS data set under analysis, realizing synergies between the two conceptually different approaches to library-free DIA analysis, spectrum-centric conversion via, e.g., DIA-Umpire^8^ and *in silico* prediction via, *e.g.*, Prosit.^10^

We implemented this approach in an R package, MSLibrarian, that supplements the Prosit framework to generate DIA-refined and trimmed spectral libraries with up to 13% improved performance as demonstrated using internal and external ground truth data sets and across two popular tools for DIA-MS data analysis. We demonstrated that library optimizations transcend into improved peptide-centric DIA-MS analysis results on the levels of both, identification sensitivity (absolute numbers of identified analytes at matched *q*-value cutoff), as well as improved quantification as assessed by multiple quality metrics. Selection of variable CE parameters for spectrum prediction proved beneficial only in one of the two data sets. As an alternative route, we demonstrated the utility and performance of a simplified workflow employing spectra predicted with static CE parameters in combination with the downstream optimization modules. Although MSLibrarian has been tested for DIA-MS data sets generated via nanoflow liquid chromatography and high-resolution Orbitrap mass spectrometry, we expect these benefits to extend to other DIA-enabled LC-MS instrumentation and software not covered in our evaluations. MSLibrarian leverages synergies between spectrum-centric and prediction-based library-free analysis approaches to facilitate deeper and more accurate mining of DIA-MS data maps and should find wide application in the field of DIA-based proteomics. It is implemented in the popular R framework for easy usage and extensibility by the DIA proteomics community and made available at https://github.com/MarcIsak/MSLibrarian.

## Supporting Information Available

Figure S1, details of the MSLibrarian workflow in R (related to Figure 1, providing a detailed view of the modules and functions of the MSLibrarian R package); Figure S2, distributions of protein group log2 fold change between samples A and B for each library in the mouse-yeast mixture data set (related to Figure 2); Figure S3, distributions of protein group log2 fold change between conditions for each library in the external multispecies mixture data set (related to Figure 3 and Figure S2, displaying *xy* scatter and density distributions of species log2 fold changes observed between conditions and further illustrating the processing time in DIA-NN required per library); Figure S4, benchmarking of simplified MSLibrarian workflow with fixed collision energy setting (related to Figure 2 and Figure 3, performance estimation by gained valid ratios and overall identifications for libraries with constant collision energy settings as in the standard Prosit workflow, optimized only with the downstream protein set and transition filters of MSLibrarian); Figure S5, impact of MSLibrarian optimizations on scores in downstream peptide-centric analysis (related to Figure 3, exploring impact of DeepLC-based retention time prediction and collision energy optimization on delta RT and spectrum similarity scores in EncyclopeDIA); and MSLibrarian user instructions (PDF)
Table S1, quantitative tables underlying library performance benchmarks (XLSX)

## Notes

Raw data, peptide-centric analysis results as well as spectral warehouse databases, spectral libraries, and protein sequence databases have been uploaded to the ProteomeXchange Consortium via the Pride partner repository (accession number: PXD028901).

## Abbreviations

DIA: data-independent acquisition
DDA: data-dependent acquisition
CE: collision energy
LC: liquid chromatography
