# Supplementary material for: MSLibrarian: Optimized Predicted Spectral Libraries for Data-Independent Acquisition Proteomics
Source: J Proteome Res. 2022 Jan 19;21(2):535–46. doi: 10.1021/acs.jproteome.1c00796 (PMC8822486; doi:10.1021/acs.jproteome.1c00796)
Supplement: Supplementary file 1 — pr1c00796_si_001.pdf [file pr1c00796_si_001.pdf]

Supporting information for

MSLibrarian: Optimized predicted spectral libraries for DIA proteomics

Marc Isaksson[1,2,*], Christofer Karlsson[3], Thomas Laurell[1] and Agnete Kirkeby[2,4,5], Moritz Heusel[3,*]

[1] Department of Biomedical Engineering, Lund University, 22100 Lund, Sweden

[2] Department of Experimental Medical Science and Wallenberg Center for Molecular Medicine, Lund University, 22100 Lund, Sweden

[3] Infection Medicine Proteomics Lab, Division of Infection Medicine (BMC), Faculty of Medicine, Lund University, 22100 Lund, Sweden

[4] Department of Neuroscience, University of Copenhagen, DK-2200 Copenhagen, Denmark.

[5] The Novo Nordisk Foundation Center for Stem Cell Biology (DanStem), Faculty of Health and Medical Sciences, University of Copenhagen, DK-2200 Copenhagen, Denmark.

* Corresponding authors (marc.isaksson@bme.lth.se, moritz.heusel@med.lu.se)

Table of Contents

Supplementary Figures S1-S5 2

MSLibrarian user instructions 8

Refinement of predicted spectral libraries with MSLibrarian 8

Brief background 8

Create a Calibration Library 8

Process the Calibration Library 9

Create the predicted spectral library 9

Make modified libraries with *mod.spectral.lib()* 10

Protein group subsetting 10

Transition subsetting 11

Retention time replacement 11

Generating customized spectral warehouse database using user-defined protein sequence databases and the Prosit web service 12

Background 12

Make Prosit input files (*.csv) for upload to the Prosit server 12

Upload prosit input files to prediction server 13

Create the SQLite database 18

Supplementary Figures S1-S5

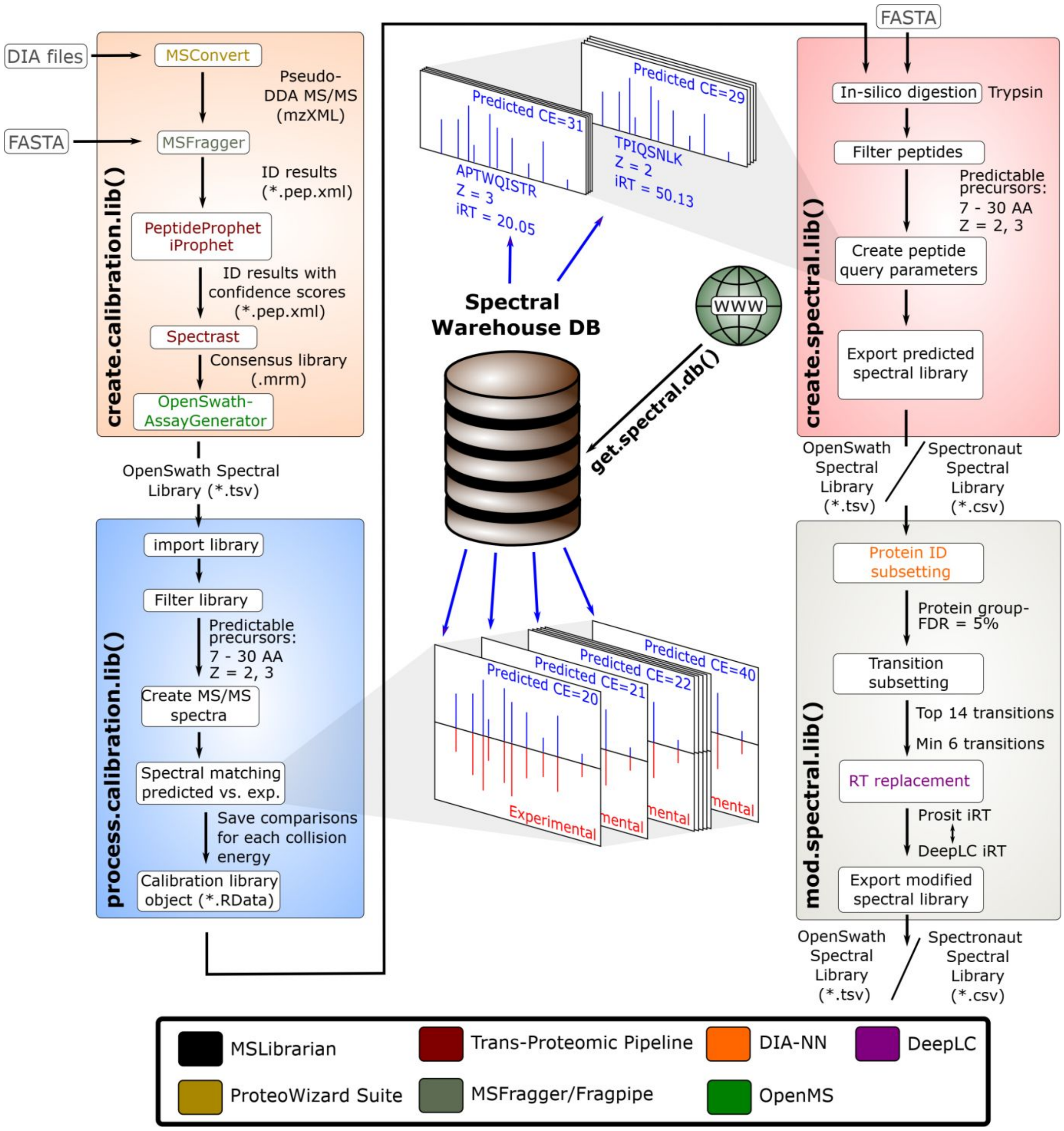

Figure S1. Details of the MSLibrarian workflow in R. Function *create.calibration.lib*: A set of DIA-MS files are converted into pseudo-DDA MS/MS spectra with MSConvert and its implementation of the DIA Umpire signal extraction module. Spectra are then searched against a user-provided protein sequence database using MSFragger and identifications validated by PeptideProphet and iProphet and a calibration library is generated via Spectrast. Function *process.calibration.lib*: The MS/MS spectra in the spectral library are then compared against predicted MS/MS spectra in a Spectral warehouse database (SQLite DB containing Prosit-predicted MS/MS spectra) using the dotproduct score to determine per each precursor length- and charge class the CE value yielding predicted spectra with highest similarity to the observed spectra in the calibration library. Function *create.spectral.lib*: A new

spectral library is created from an in-silico digested FASTA, with fragment ion intensities extracted from the Spectral warehouse database, initially using retention times as predicted by Prosit. Function *mod.spectral.lib*: Downstream modification of predicted spectral libraries; including Protein ID subsetting (e.g. from First-pass analysis in DIA-NN, or the spectrum-centric search results); Transition subsetting and replacement of Prosit iRT values with DeepLC-predicted iRT values that have been calibrated with iRT values from the calibration library.

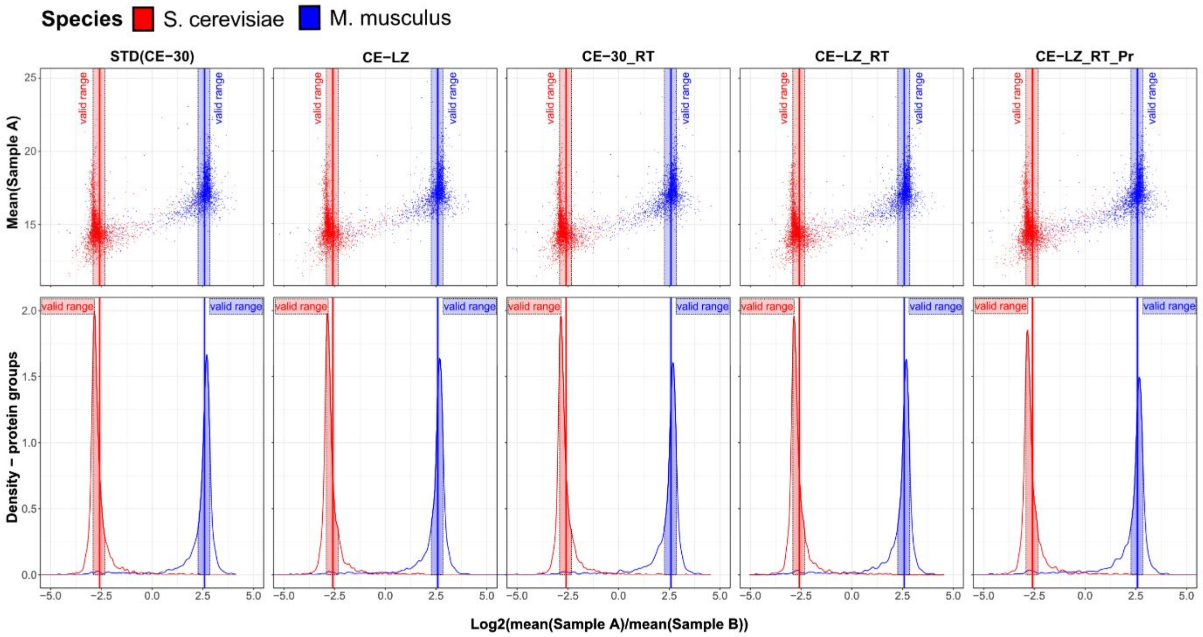

Figure S2. Distributions of protein group log2 fold change between Sample A and Sample B for each library in the Mouse:Yeast mixture dataset. The upper panel shows the fold changes for each individual protein group as a function of log2 intensity (In sample A), while the lower panel shows the projected density distributions of the protein group fold changes. The filled areas in blue and red mark the fold changes considered valid, within ± 20 % tolerance from the theoretical fold changes (solid vertical lines).

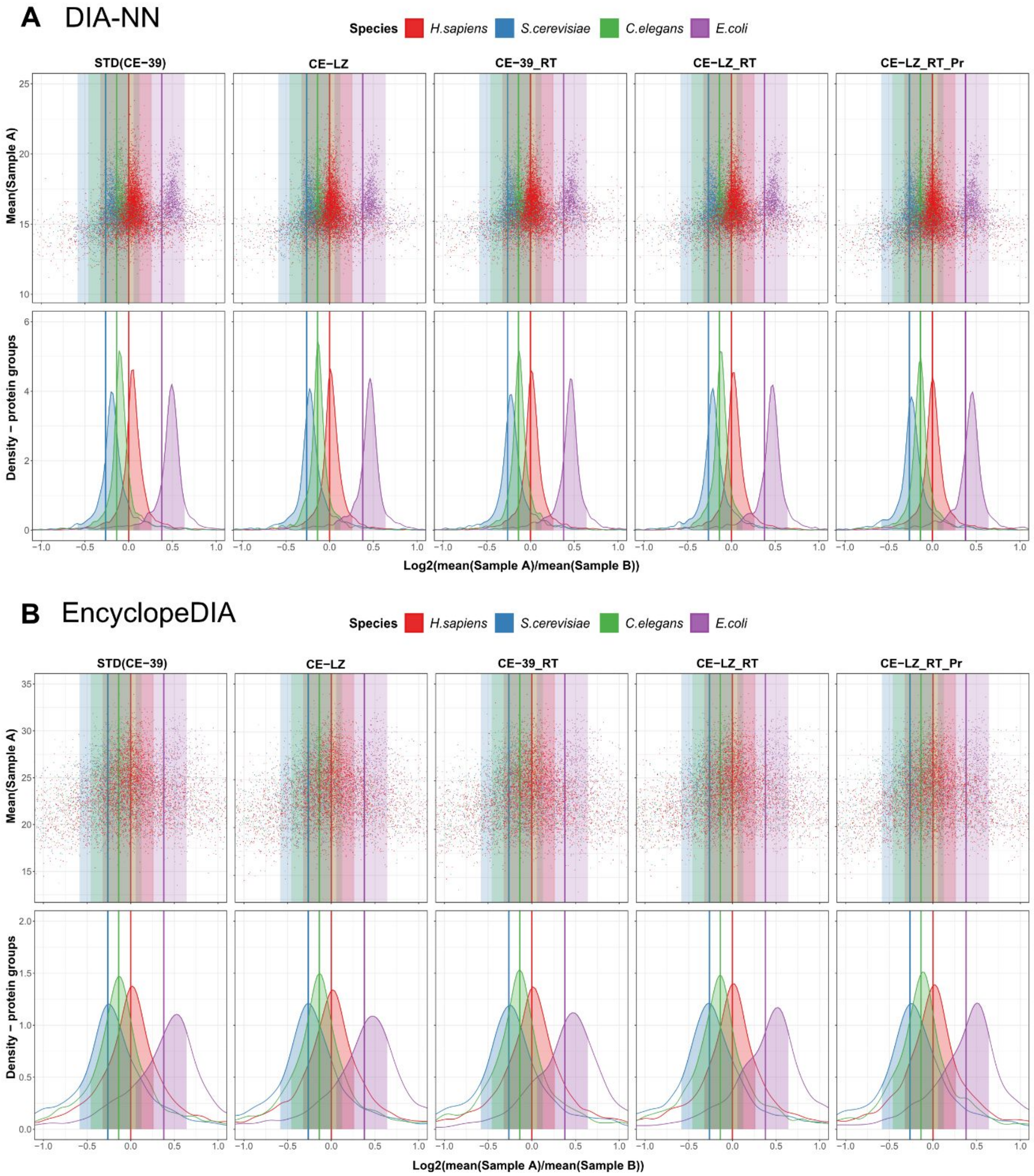

Figure S3. Distributions of protein group log2 fold change between conditions for each library in the external multi-species mixture dataset. **A** Protein group fold change distributions for the external dataset (PXD005573) from the mixed species samples when analysed in DIA-NN 1.8, separated for each predicted library. In the upper panel, the fold change for each protein group is displayed. In the lower panel, the protein group fold change densities are shown. Valid fold change ranges, allowing for a ± 20 % deviation from the theoretical fold change, are indicated by shaded areas. True mixing ratio fold-changes are shown by solid vertical lines per species. **B** Equivalent to panel A, albeit when DIA data are analysed via EncyclopeDIA 1.2.2_win, using standard parameters and protein quantification settings (Materials and Methods). In the upper panel, the fold change for each protein group is

displayed. In the lower panel, the protein group fold change densities are shown. Valid fold change ranges, allowing for a ± 20 % deviation from the theoretical fold change, are indicated by shaded areas. True mixing ratio fold-changes are shown by solid vertical lines per species.

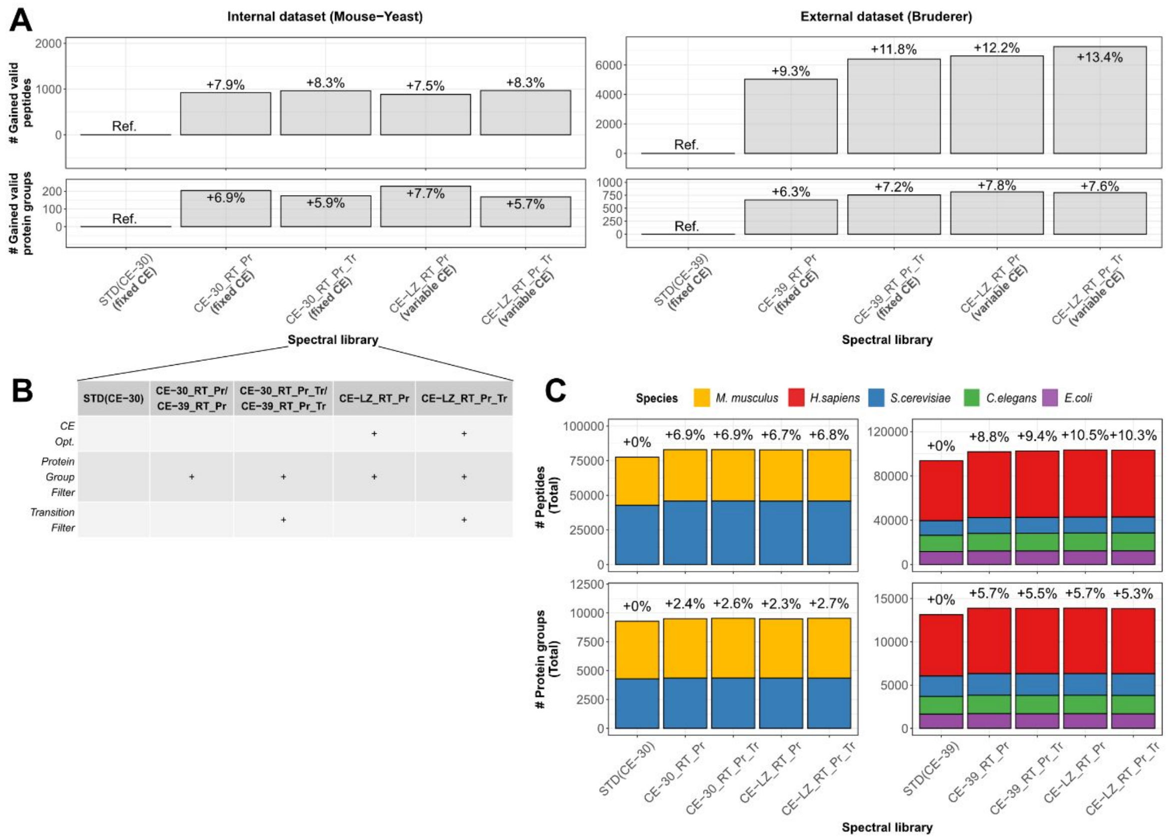

Figure S4. Benchmarking of simplified MSLibrarian workflow with fixed collision energy setting. **A** Peptide-level (upper panels) and protein-level (lower panels) performance in ratio conformance benchmark of fixed-CE libraries vs. variable-CE libraries in Mouse:Yeast dataset (left panels) and Bruderer et al. dataset (right panels). Fixed CE libraries with optimization of protein set (_Pr) and in combination with transition selection (_Pr_Tr) are contrasted against the corresponding variable CE libraries (CE-LZ_) and the standard fixed CE library as displayed also in Figure 2 and Figure 3. **B** Overview scheme of processing steps applied to generate each of the libraries compared here. **C** Total peptide and protein group identifications obtained at 1 % q-value cutoff on peptide and protein level (global scale) upon targeted analysis with either of the libraries compared here.

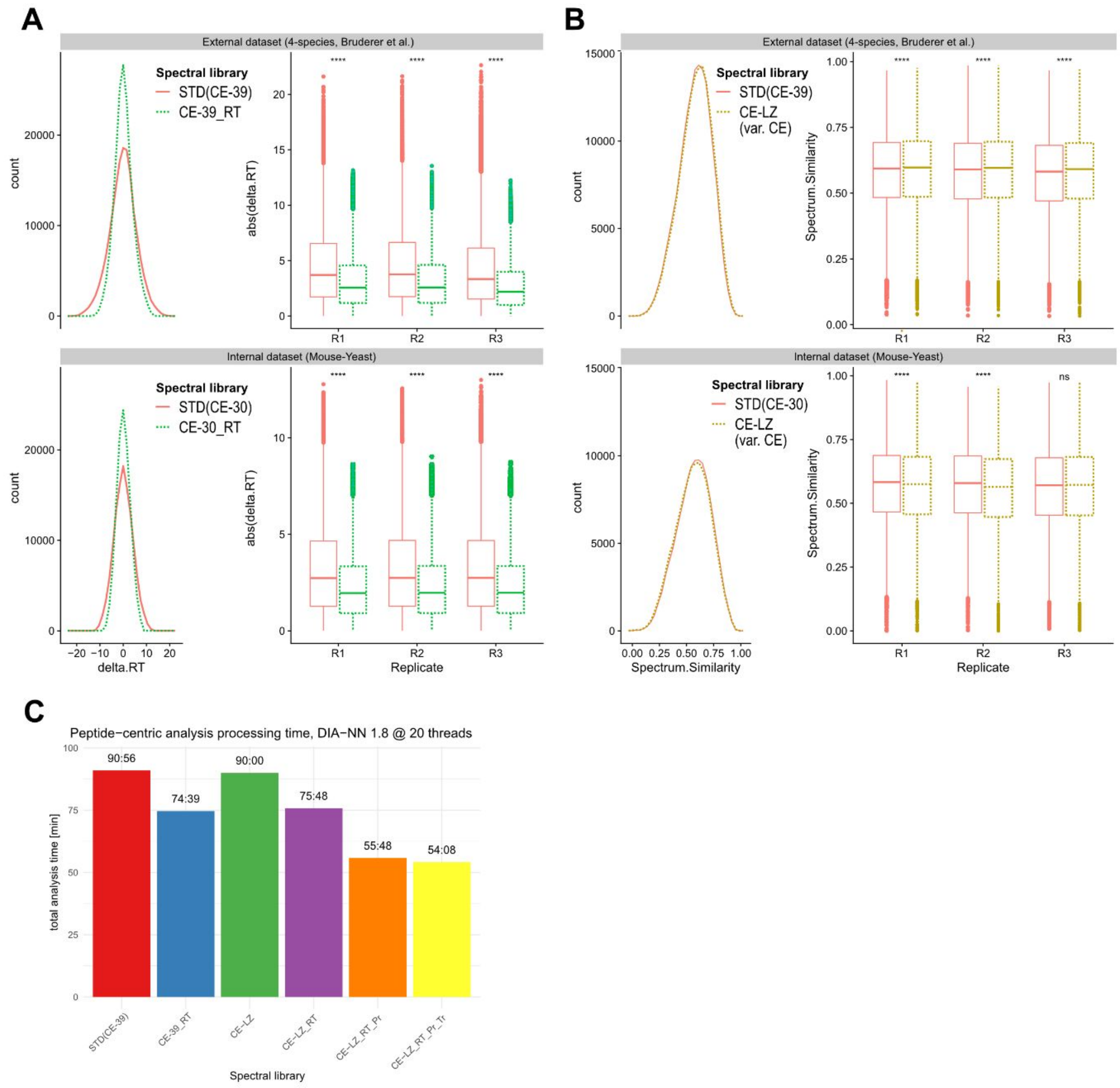

Figure S5. Impact of MSLibrarian optimizations on scores in downstream peptide-centric analysis. EncyclopeDIA retention time scoring analysis on both datasets (Upper panels, external, lower panels, internal dataset as indicated). **A** Retention time re-calibration via DeepLC (Libraries '_RT') leads to sharper delta.RT score distributions (left, density plot across replicates) and significantly lower absolute delta.RT readings consistently across replicates and both datasets (boxplots in right panels, unpaired t.test p < 10e-4 in all 3 replicates of both datasets). **B** Variable CE selection, comparing the library CE-LZ vs. the respective standard, fixed CE library. Bruderer et al. dataset shows significantly higher Spectrum.Similarity scores globally (left) and consistently across replicates (boxplots, right, unpaired t.test p < 10e-4 in all 3 replicates). Lower panels: For the Mouse-Yeast dataset, Spectrum.Similarity appears globally lower (left) and is affected negatively in two out of three replicates, and insignificanly higher in the third replicate (boxplots, right, unpaired t.test p < 10e-4 in for similarity reduction in replicates 1 and 2). C Assessment of peptide-centric analysis processing times with the different libraries, using 20 threads on a Intel(R) Core(TM) i9-7900X CPU @ 3.30GHz. Up to 40 % reduction on processing time is observed (54:08 min, CE-LZ_RT_Pr_Tr, vs. 90:56 min for library STD(CE-39)).

MSLibrarian user instructions

Refinement of predicted spectral libraries with MSLibrarian

Brief background

In this wiki, we will use MSLibrarian to create a predicted spectral library. For demonstration purposes, the spectral library will be created to analyse DIA runs of Yeast samples (*Saccharomyces cerevisiae*).

Create a Calibration Library

First, we begin by creating a Calibration Library that will be used to extract optimal predicted fragment ion intensities and calibrate iRT predictions.

Preparations

First we start by finding the paths to the DIA runs.

```
diaFolder = "Y:/imp_bioms/CK/mslibr_test/YeastDIA/"  # The folder with DIA MS files in RAW format
diaFiles = list.files(diaFolder, pattern = ".raw$", full.names = T) # Extracts the full paths to all raw files
diaFiles # prints the file names.`

"Y:/imp_bioms/CK/mslibr_test/YeastDIA/CK_P2005_306.raw"
"Y:/imp_bioms/CK/mslibr_test/YeastDIA/CK_P2005_307.raw"
"Y:/imp_bioms/CK/mslibr_test/YeastDIA/CK_P2005_308.raw"
"Y:/imp_bioms/CK/mslibr_test/YeastDIA/CK_P2006_079.raw"
"Y:/imp_bioms/CK/mslibr_test/YeastDIA/CK_P2006_080.raw"
"Y:/imp_bioms/CK/mslibr_test/YeastDIA/CK_P2006_085.raw"
```

In the second step, we define some parameters to use for the first function:

```
projectFolder = "D:/demo_mslibr_yeast" # Project folder to save all MSLibrarian outputs into
fasta = "D:/Databases/Uniprot_Swissport_Yeast_210710/Canonical_Isoforms/uniprot_swissprot_yeast.fasta" # Path to protein sequence FASTA
searchEngine = "msfragger" # Database search engine to use (default = "comet")
irt = "biognosys_irt" # Use Biognosys iRT values as RT scale. If argument is not provided, retention times will be in seconds.
```

Run create.calibration.lib()

To create the Calibration Library, we run the following MSLibrarian function:

```
create.calibration.lib(projectFolder = projectFolder,
                       fasta = fasta,
                       diaFiles = diaFiles,
```

```
                        searchEngine = searchEngine,
                        irt = irt)
```

Once the the function execution has completed, there should be a Calibration Library in OpenSwath (*tsv) format, located in the Project folder that you created (“D:/demo_mslibr_yeast/library/calibration_lib.tsv”)

Process the Calibration Library

Once a Calibration Library has been created, it is possible to compare experimental spectra in the Calibration Library to spectra predicted by Prosit at different collision energies. In this way, the optimal Prosit collision energies can be determined for extracting predicted fragment ion intensities that are most similar to experimental intensities.

Setting up parameters

Before comparing experimental spectra to predicted spectra, we need to specify some new parameters.

```
predictionDb = "D:/Data_PROSIT/Libraries/Yeast/210710/SQLITE/yeast_prosit_hcd_intensity_2020_irt_2019.sqlite" # Path to Prosit prediction SQLite DB
rt = "iRT" # The type of retention time scale to use for the latter library building
```

Run process.calibration.lib()

To run comparisons between experimental spectra and predicted spectra, we run the following function:

```
process.calibration.lib(projectFolder = projectFolder,
                        predictionDb = predictionDb,
                        rt = rt)
```

After executing the function, two more files can be found in the subfolder *library* of the project folder (“D:/demo_mslibr_yeast/library/”) * calibration_lib.RData –> contains results for similarity comparisons between experimental and predicted spectra. * calibration_lib.pdf –> Plot showing the optimal collision energies, dot products and distributions for precursors of different lengths and charges

Name	Date modified	Type	Size
calibration_lib.tsv	2021-08-10 17:28	TSV File	85 759 KB
calibration_lib.RData	2021-08-10 18:47	R Workspace	15 237 KB
calibration_lib.pdf	2021-08-10 18:47	Adobe Acrobat D...	14 KB

Create the predicted spectral library

Since the optimal collision energies have been determined for extracting predicted fragment ion intensities, we can build a predicted spectral library. The library in this case will have both fragment ion intensities and iRT values predicted by Prosit.

To run create the library, we only need to specify one new parameter:

```
format = "openswath" # Spectral library output format
```

To create the spectral library we run the following MSLibrarian function:

```
create.spectral.lib(projectFolder          =          projectFolder,
                    fasta                  =                  fasta,
                    format = "openswath")
```

After running the function, a predicted spectral library can be found in the subfolder `library` of the project folder ("D:/demo_mslibr_yeast/library/). Since we did not define an output name (can be set with the argument *outputLib*), an output name is generated automatically for us. The library name contains information about the date and time for its creation, but also information on the strategy for selecting fragment ion intensities (**ceMode** = **length_charge** by default) and the algoritm for prediction of iRT values (**_irt_prosit**).

Name	Date modified	Type	Size
calibration_lib.tsv	2021-08-10 17:28	TSV File	85 759 KB
calibration_lib.RData	2021-08-10 18:47	R Workspace	15 237 KB
calibration_lib.pdf	2021-08-10 18:47	Adobe Acrobat Document	14 KB
Aug_11_09_39_30_2021_mslibrarian_ce_length_only_irt_prosit.tsv	2021-08-11 09:43	TSV File	986 969 KB

Make modified libraries with *mod.spectral.lib()*

Once a spectral library has been created, several modified versions of that library can be created. In this section, we will go through how to create spectral libraries that have been subsetted on the protein group level, peptide level and transition level. Finally, we will see how retention times can be changed from Prosit iRT to DeepLC iRT.

Protein group subsetting

Protein group subsetting of a library can be performed in 3 different ways. Either the DIA software DIA-NN can be used to perform a first-pass search of the unmodified library, followed by the extraction of protein groups passing a FDR threshold of for example 5% (arg - **protFDR**). The protein groups passing the FDR threshold will then be used to subset the unmodified library, and the subsetted library can then be used for a second-pass search. Another option is to subset the unmodified library, using the identified protein groups in the

Calibration Library built using *MSLibrarian*. The third option is to include a character vector of protein accession numbers (Uniprot) to directly filter the library. This approach could be beneficial to

To perform protein group subsetting with DIA-NN, we define the following parameters:

```
inputLib = "D:/demo_mslibr_yeast/library/Aug_11_10_20_32_2021_mslibrarian_ce_length_charge_irt_prosit.tsv" # input spectral library
mods = c("protein") # Modifies the library on the protein group level
protMod = "diann" # Type of protein modification.
protFdr = 0.01 # Protein group FDR to use to subset the input library
diannPath = "C:/Program Files/DIA-NN_1.8/DiaNN.exe"
```

To subset the library on protein level, run `MSLibrarian::mod.spectral.lib()`:

```
mod.spectral.lib(projectFolder = projectFolder,
                 inputLib = inputLib,
                 diaFiles = diaFiles,
                 mods = mods,
                 protMod = protMod,
                 protFdr = protFdr,
                 diannPath = diannPath)
```

Transition subsetting

Predicted libraries may contain many transitions with relative intensities close to zero. There may also be targets/precursors in the library that has very few transitions which makes MS2-based quantification difficult. With the function`mod.spectral.lib()` it is possible to apply different transition filters to subset a library.

In this example, we will set the following parameters to perform transition filtering:

```
mods = c("transition") # Modify spectral library on transition level
topTrans = 14 # Maximum 14 transitions per library target/precursor
cutoffTrans = 0.01 # Minimum relative intensity for a transition
minTrans = 6 # Minimum transitions that a library target/precursor must have
```

To create a transition-subsetted library we run:

```
mod.spectral.lib(projectFolder = projectFolder,
                 inputLib = inputLib,
                 mods = mods,
                 topTrans = topTrans,
                 cutoffTrans = cutoffTrans,
                 minTrans = minTrans)
```

Retention time replacement

MSLibrarian allows for the replacement of retention times in a library from the default Prosit iRT to either DeepLC iRT or RT(minutes). A benefit of using DeepLC over Prosit, is the ability to calibrate the retention time predictions with peptides of known retention times, which

may increase the prediction accuracy. When carrying out retention time replacement with MSLibrarian, a small proportion of peptides of known retention times will be extracted from the **Calibration Library** that is created in the beginning. These peptides are then used to calibrate DeepLC predictions of either iRT or RT values for each target in the library.

To replace retention times in a library, there is only one parameter that needs to be specified.

```
mods = c("rt")
```

Then we simply run `MSLibrarian::mod.spectral.lib()`:

```
mod.spectral.lib(projectFolder = projectFolder,
                 inputLib = inputLib,
                 mods = mods)
```

A new library with replaced retention times is then outputted (Aug_11_10_20_32_2021_mslibrarian_ce_length_charge_rt_deeplc_0.25.tsv). Apart from the library, a figure is outputted which shows the correlation between experimental iRTs for all targets in the **Calibration Library** and the corresponding DeepLC-predicted iRT values.

Name	Date modified	Type	Size
Aug_11_10_20_32_2021_mslibrarian_ce_length_charge_rt_deeplc_0.25.tsv	2021-08-12 08:41	TSV File	991 217 KB
Aug_11_10_20_32_2021_mslibrarian_ce_length_charge_irt_prosit.tsv	2021-08-11 10:24	TSV File	967 135 KB

Type `?mod.spectral.lib` in the R Console to read the documentation on the `mod.spectral.lib`

Generating customized spectral warehouse database using user-defined protein sequence databases and the Prosit web service

Background

In this wiki, we will go through the necessary steps to create our very own Spectral Warehouse Database in SQLite format from Prosit predictions. These databases can be used to quickly create Prosit-predicted spectral libraries in MSLibrarian without having to submit jobs to a prediction server every time, or bother installing Prosit locally on a machine with a CUDA-enabled graphics card. As a demonstration, we will create a SQLite database for the the entire reviewed proteome of *Drosophila Melanogaster* (Fruit fly) that has been downloaded as a FASTA from Uniprot/Swissprot.

Make Prosit input files (*.csv) for upload to the Prosit server

Preparations

To create a SQLite database, we begin by defining some parameters:

```
fasta =
"D:/Databases/Uniprot_Swissprot_FruitFly_210806/uniprot_swissprot_drosophila_melanogaster_210806.fasta"
chargeRange = c(2,3) # Allowed precursor charges. DO NOT CHANGE THIS!
ceRange = c(20,40) # Set the range of Prosit collision energies for which
```

```
fragment ion intensities will be predicted
prefix = "drosophila_melanogaster" # Prefix of input CSV files to be uploaded
to the Prosit online tool.
outputFolder = "D:/Data_PROSIT/Input/Fruitfly/210806/" # Path to folder
where Prosit input files will be added.
```

Create Prosit input files

To create input files for the Prosit prediction server, we run the function `MSLibrarian::make.prosit.csv()` after adding the above specified parameters as arguments. Apart from the Prosit input files, a RData file is added to the output folder with metadata for the SQLite database to be created.

```
library(MSLibrarian)
make.prosit.csv(fasta = fasta, chargeRange = chargeRange, ceRange = ceRange,
prefix = prefix, outputFolder = outputFolder)

[1] "Load FASTA..."\
[1] "Importing FASTA database:
D:/Databases/Uniprot_Swissprot_FruitFly_210806/uniprot_swissprot_drosophil
a_melanogaster_210806.fasta..."\
[1] "Database contains: 5058 proteins..."\
[1] "Adding protein data to slot: Proteins of the MSLibrarian object..."\
[1] "Assuming all Cysteines are Carbamidomethylated"\
[1] "In-silico digestion of proteins into peptides using trypsin"\
[1] "Calculating masses..."\
[1] "Done!"\
[1] "Filter peptides..."\
[1] "Finding duplicated peptides..."\
[1] "Creating precursor data..."\
[1] "Removing duplicated sequences..."\
[1] "Calculating m/z values..."\
[1] "Number of unique precursors: 200100"\
[1] "Number of unique predictable precursors within M/Z range (0 - Inf):
200100"\
[1] "Preparing for writing files..."\
[1] "writing: drosophila_melanogaster_digest_ce20.csv"\
[1] "writing: drosophila_melanogaster_digest_ce21.csv"\
[1] "writing: drosophila_melanogaster_digest_ce22.csv"\
[1] "writing: drosophila_melanogaster_digest_ce23.csv"\
[1] "writing: drosophila_melanogaster_digest_ce24.csv"\
[1] "writing: drosophila_melanogaster_digest_ce25.csv"\
[1] "writing: drosophila_melanogaster_digest_ce26.csv"\
[1] "writing: drosophila_melanogaster_digest_ce27.csv"\
[1] "writing: drosophila_melanogaster_digest_ce28.csv"\
[1] "writing: drosophila_melanogaster_digest_ce29.csv"\
[1] "writing: drosophila_melanogaster_digest_ce30.csv"\
[1] "writing: drosophila_melanogaster_digest_ce31.csv"\
[1] "writing: drosophila_melanogaster_digest_ce32.csv"\
[1] "writing: drosophila_melanogaster_digest_ce33.csv"\
[1] "writing: drosophila_melanogaster_digest_ce34.csv"\
[1] "writing: drosophila_melanogaster_digest_ce35.csv"\
[1] "writing: drosophila_melanogaster_digest_ce36.csv"\
```

```
[1]             "writing:           drosophila_melanogaster_digest_ce37.csv"\
[1]             "writing:           drosophila_melanogaster_digest_ce38.csv"\
[1]             "writing:           drosophila_melanogaster_digest_ce39.csv"\
[1]             "writing:           drosophila_melanogaster_digest_ce40.csv"\
[1]"Saving                         SQLite                           metadata
to:D:/Data_PROSIT/Input/Fruitfly/210806//drosophila_melanogaster_metadata.
RData"
```

Upload prosit input files to prediction server

Go to the Prosit online tool: https://www.proteomicsdb.org/prosit/ and click on the **PREDICT** tab and then select the **SPECTRAL LIBRARY** tab. After reading through the information under Settings, click on the **NEXT** tab.

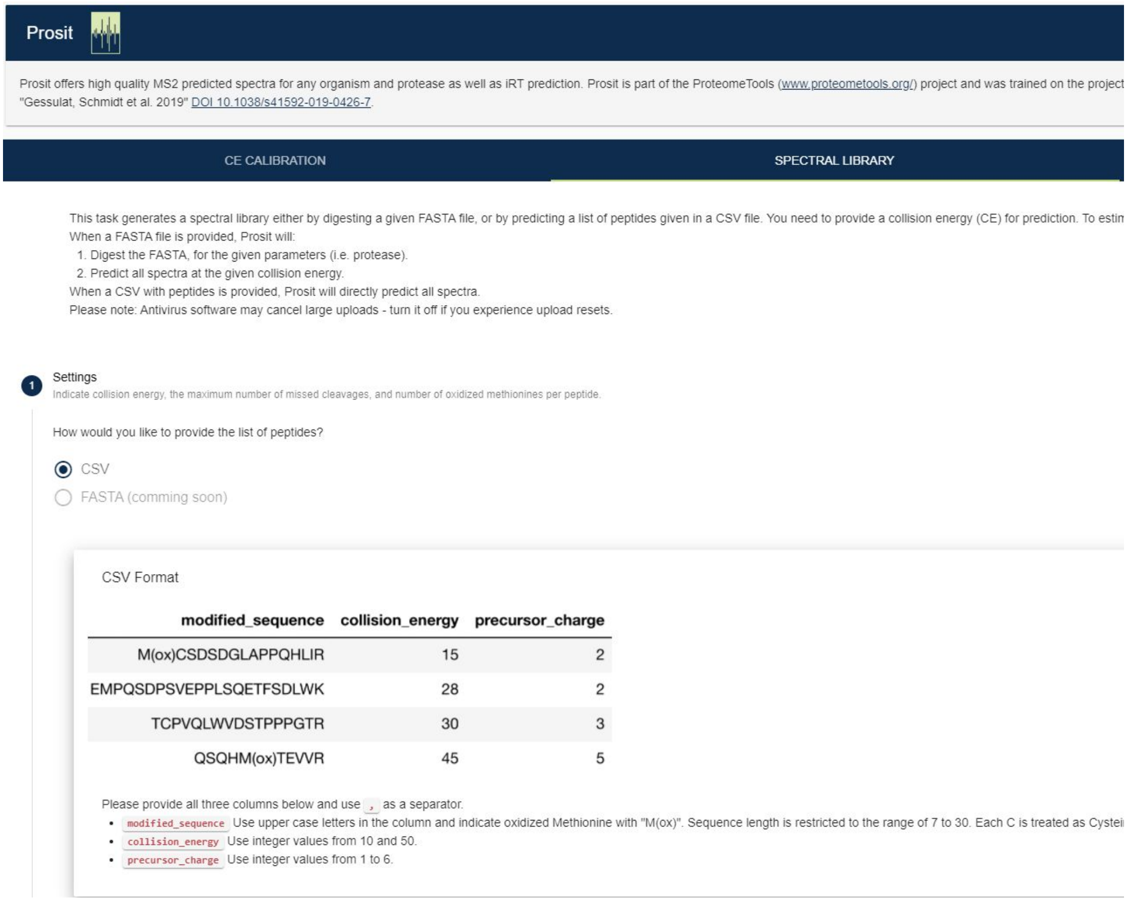

Then click on the blue upload button and navigate to the folder where the input files were added: `D:/Data_PROSIT/Input/Fruitfly/210806/`

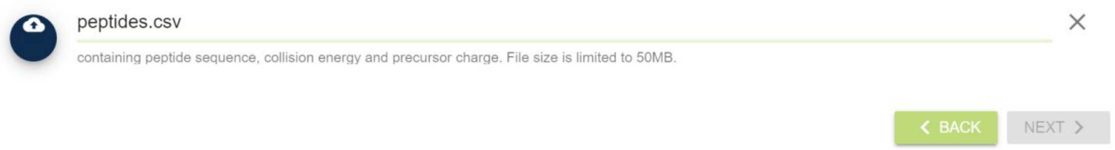

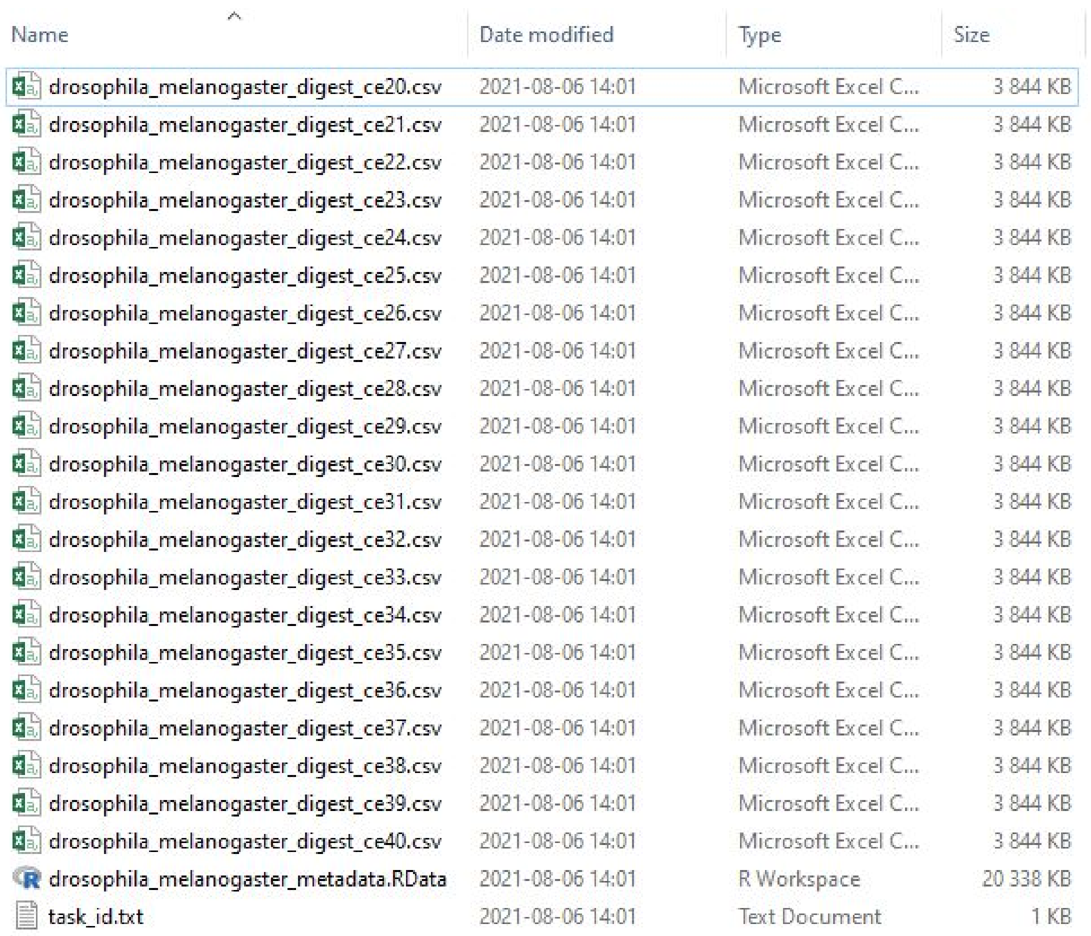

Select the first Prosit input file (**drosophila_melanogaster_digest_ce20.csv**) and click **Open** to upload it. Once uploading is complete, click on the **Next** tab

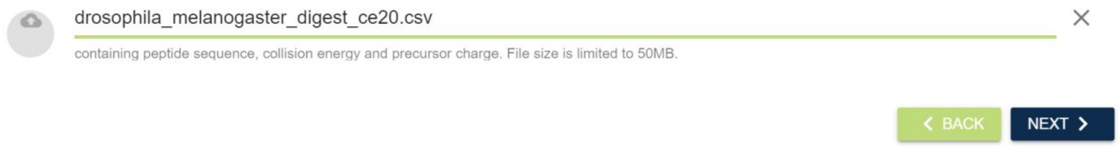

Under **Model** choose *Prosit_2020_intensity_hcd* as intensity prediction model (or choose *Prosit_2020_cid* if your instrument method used CID for fragmentation). For iRT prediction model select *Prosit_2019_irt* and then click **NEXT**.

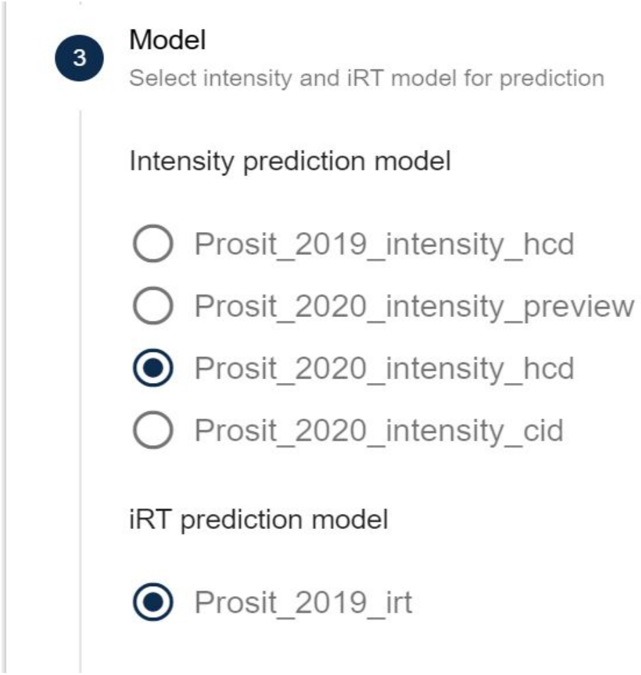

Under TASK ID choose the format **Generic text (Spectronaut compatible)** format. Do not choose the **MSP**-format. It is incompatible with MSLibrarian.

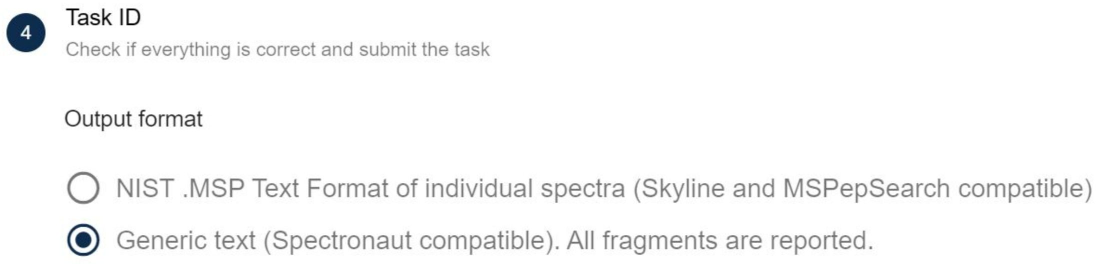

To submit a job to the prediction server, click on the **SUBMIT**-button. A Task ID for the submitted job will be shown in the next window.

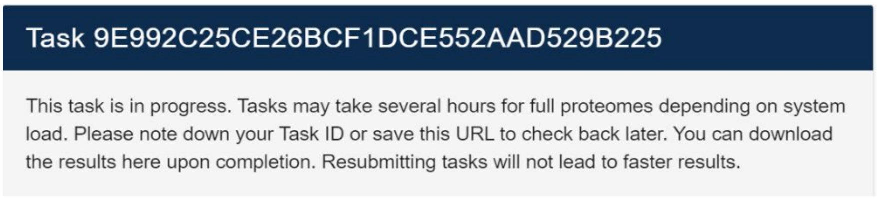

Copy the Task ID and then open the *task_id.txt* file located in the folder with Prosit input files (D:/Data_PROSIT/Input/Fruitfly/210806/task_id.txt). Next to the name of the submitted Prosit input csv file, paste and replace the NA value with the Task ID that you copied.

```
task_id.txt - Notepad
File  Edit  Format  View  Help
pred_file       task_id
drosophila_melanogaster_digest_ce20.csv 9E992C25CE26BCF1DCE552AAD529B225
drosophila_melanogaster_digest_ce21.csv NA
drosophila_melanogaster_digest_ce22.csv NA
drosophila_melanogaster_digest_ce23.csv NA
drosophila_melanogaster_digest_ce24.csv NA
drosophila_melanogaster_digest_ce25.csv NA
drosophila_melanogaster_digest_ce26.csv NA
drosophila_melanogaster_digest_ce27.csv NA
drosophila_melanogaster_digest_ce28.csv NA
drosophila_melanogaster_digest_ce29.csv NA
drosophila_melanogaster_digest_ce30.csv NA
drosophila_melanogaster_digest_ce31.csv NA
drosophila_melanogaster_digest_ce32.csv NA
drosophila_melanogaster_digest_ce33.csv NA
drosophila_melanogaster_digest_ce34.csv NA
drosophila_melanogaster_digest_ce35.csv NA
drosophila_melanogaster_digest_ce36.csv NA
drosophila_melanogaster_digest_ce37.csv NA
drosophila_melanogaster_digest_ce38.csv NA
drosophila_melanogaster_digest_ce39.csv NA
drosophila_melanogaster_digest_ce40.csv NA

Ln 2, Col 73    100%    Unix (LF)    UTF-8
```

Repeat the steps above to submit the remaining 20 Prosit input CSV (drosophila_melanogaster_digest_ce21.csv, _22.csv, …,_40.csv). Thus, the Task ID text file (task_id.txt) should look similar to the example below once all jobs have been submitted to the prediction server.

```
task_id.txt - Notepad
File  Edit  Format  View  Help
pred_file       task_id
drosophila_melanogaster_digest_ce20.csv 9E992C25CE26BCF1DCE552AAD529B225
drosophila_melanogaster_digest_ce21.csv AF100A818515E108D054E8FE2504C6EF
drosophila_melanogaster_digest_ce22.csv CD632D0D37EE6DE4931523FC842F0271
drosophila_melanogaster_digest_ce23.csv 4390D78019D061B40F7DA42A9DBBE4E5
drosophila_melanogaster_digest_ce24.csv 808D24AC2762118B014DF17E3B36BF40
drosophila_melanogaster_digest_ce25.csv 2F1B1878FAEDB19E0F8D9E2D66BEBCED
drosophila_melanogaster_digest_ce26.csv 54ED44FA863DD4E6AEB03F9BFEC6BF57
drosophila_melanogaster_digest_ce27.csv 71B53FCEE7871CAAD1AD9D1AD38A0870
drosophila_melanogaster_digest_ce28.csv 385F85651E8E51E91AEBCB2A784AFFD6
drosophila_melanogaster_digest_ce29.csv A10893BDC970561A7733B2DC5F7607E0
drosophila_melanogaster_digest_ce30.csv 3E00314EC062461A3473C064BE0AB991
drosophila_melanogaster_digest_ce31.csv 86AC94E563B18877318B35679BD6CD6B
drosophila_melanogaster_digest_ce32.csv 4E760DEE8C69CD8BE328A4A32D684B1B
drosophila_melanogaster_digest_ce33.csv CBFA644D86462738E4E358DED85431EA
drosophila_melanogaster_digest_ce34.csv 793AD6CB10E41BC73451F4E3698D6EE8
drosophila_melanogaster_digest_ce35.csv DFFDFA11A2B368E8D11501E4EF990FAA
drosophila_melanogaster_digest_ce36.csv 33A0CCFE2FF774AE3AF7B0EF6069A902
drosophila_melanogaster_digest_ce37.csv 616644AD73DD0AFD425E11F7DB26B0EF
drosophila_melanogaster_digest_ce38.csv 9A2C192321D3B3A2F1A03B0C25138D48
drosophila_melanogaster_digest_ce39.csv 7DCB4DD044B0679A878144F30B3EA79A
drosophila_melanogaster_digest_ce40.csv DFF3BCBC0052B9B94FCEB1AB3AA93411

Ln 1, Col 1    100%    Unix (LF)    UTF-8
```

Save the *task_id.txt* file.

The prediction of spectral libraries for entire proteomes may take between 1 to 2 days to complete. In order to be sure that all predictions are made, it is recommended to wait 2 days before continuing building the SQLite database. Alternatively, the Task ID of the last submitted job may be checked on the Prosit website (https://www.proteomicsdb.org/prosit/). To do this, click on the **STATUS** tab in the top right corner and paste the Task ID of the last submitted job.

If the job is completed, there should be a message on the next page saying: Your files are ready! (NB! Do not click the **DOWNLOAD** button) If this is the case, all previous jobs should be completed and the SQLite database can be built without any problems.

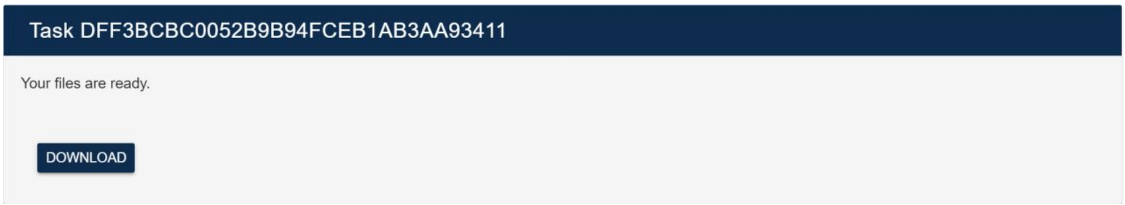

Create the SQLite database

Once all submitted prediction jobs are completed, it is possible to create the prediction SQLite database. First, we will define some parameters:

```
prediction_folder = "D:/Data_PROSIT/Libraries/Fruitfly/210806/" # The SQLite subfolder with the SQLite database (*.sqlite) will be added to this folder
task_id = "D:/Data_PROSIT/Input/Fruitfly/210806/task_id.txt" # absolute path to the Task ID file
sqlite = "drosophila_melanogaster_prosit_hcd_intensity_2020_irt_2019.sqlite" # Preferred name of the SQLite database"
```

Then we run the following MSLibrarian function to create the SQLite database:

```
make.prediction.db(prediction_folder = prediction_folder,
                   task_id = task_id,
                   sqlite = sqlite)
```

After completion, there should be a subfolder named SQLite in the specified `prediction_folder`. Inside the subfolder, the prediction SQLite database can be found ("drosophila_melanogaster_prosit_hcd_intensity_2020_irt_2019.sqlite")
